# Fouling release coatings reduce colonisation of coral seeding devices

**DOI:** 10.1038/s41598-025-08268-9

**Published:** 2025-07-05

**Authors:** Jose Montalvo-Proano, Florita Flores, Andrea Severati, Andrew P. Negri

**Affiliations:** Australian Institute of Marine Science, PMB No.3, Townsville, QLD 4810 Australia

**Keywords:** Coral restoration, Antifouling, Antifoulant, Great Barrier Reef, Coral reef, Competition, Recruit, Marine biology, Restoration ecology

## Abstract

The increasing frequency and severity of coral bleaching underscores the need for effective coral reef restoration programs. These initiatives include deploying coral fragments or early recruits (spat), with large-scale coral seeding success dependent on improving coral survival by minimizing competition from algae and benthic invertebrates. This study presents a *proof-of-concept* field experiment assessing the effectiveness of two commercial non-biocidal fouling release coatings (FRCs) and an FRC wax coating in reducing fouling on coral seeding devices. Ceramic devices treated with FRCs were deployed with *Acropora millepora* microfragments and monitored over 46 weeks. Coated devices experienced significantly less fouling than uncoated controls, particularly during the critical early months when coral spat are most vulnerable. The best performing coating maintained over 10 times more clear surface area than uncoated devices, providing sustained protection without affecting coral survival. Corals also successfully overgrow the coatings by trial’s end. These findings suggest that FRCs could protect smaller coral spat from overgrowth, reducing early mortality until they reach a *size escape* threshold. Such coatings also hold promise for large-scale restoration projects, coral nurseries, and aquaculture. Future research should evaluate their efficacy across diverse habitats, particularly areas with high macroalgal cover, to optimize their application in restoration strategies.

**Supplementary Information:**

The online version contains supplementary material available at 10.1038/s41598-025-08268-9.

## Introduction

The global decline of coral reefs, driven by increasing frequency and severity of climate change-induced bleaching events, has led to growing support for the development of effective coral reef restoration programs^1–3^. These programs aim to restore degraded or damaged ecosystems through a variety of interventions^2,4^including replenishment by outplanting coral fragments or early recruits (spat)^5,6^. However, the success of restoration efforts hinges on the efficiency of each phase, as well as the technical and financial capacity to implement interventions on a reef-wide scale^4,7,8^. Large-scale coral ecosystems, such as Australia’s Great Barrier Reef (GBR), will likely require extensive interventions to achieve significant positive impacts and keep pace with future environmental degradation^9^. Therefore, alongside global efforts to mitigate greenhouse gas emissions, it is essential to strategically develop effective and scalable methods that enhance coral survival in restoration programs.

Traditional restoration projects have often relied on generating clonal fragments from healthy donor colonies and attaching them to degraded reef substrate^10,11^. These fragments can be affixed directly to existing carbonate structures^12^artificial frames^13^large artificial structures^14^plastic mesh^15^ or on a variety of smaller deployment devices that rest or are attached to the natural reef substrate^16^. These restoration methods are effective for establishing populations and have been applied at scales up to approximately one hectare, though they can require substantial resources to implement^17^. The outplanting of aquarium- or nursery-generated corals attached to small seeding devices may offer a more scalable solution, especially when deployed freely from fit-for-purpose vessels^8^. Challenges for this approach include meeting the demand for sufficient biological material (fragments), ensuring device retention on suitable substratum and maximising post-deployment coral survival^5^.

The sexual production of vast numbers of coral larvae can be achieved in aquaria^18^ or by collection of wild-spawned gametes (i.e., slicks) from the sea surface following mass spawning events^19,20^massively increasing the availability of individuals for settlement onto seeding devices. This approach can also increase genetic diversity and include offspring bred or modified to be more resilient to the conditions of climate change^21^. Experimental seeding devices have been produced from a variety of materials and often incorporate features such as spikes or arms that are designed to increase retention and stability when deployed without attachment mechanisms to the substratum^22–24^. To minimise the costs of maintaining spat in culture facilities, coral seeding devices are often deployed within the first weeks after settlement, when the spat are approximately 1 mm in diameter. However, mortality is typically high over the first year, often ranging from 70 to 90%^22,25–28^. Device designs have been modified with physical features or refugia to optimise the light environment and protect from sediment deposition and grazing or predation by fish or other organisms^29^. Direct competition with benthic alga and invertebrates presents an additional hazard to the survival of small coral spat. Documented over many decades as a key driver of early natural mortality of corals, this benthic competition has also been observed in spat on artificial substrates^30^. Coral spat grow slowly in comparison to many species of filamentous and turf algae, encrusting crustose coralline algae (CCA) and colonial invertebrates including bryozoans, and it is not until spat reach the size of > 1 cm^2^referred to as their size escape threshold, that mortality rates decline^25,31^. Spat on seeding devices are therefore at risk from native benthic organisms colonising (fouling) device surfaces and overwhelming the slow growing spat^32,33^ or surrounding them to become a trap for organic matter and resulting in sediment accumulation^34^. The manual removal of macroalgae has been proposed for some localised areas to enhance coral spat survival^35–37^; however, ongoing seeding device maintenance may be prohibitive at larger scales. For seeding devices to succeed as a large-scale coral reef restoration intervention, effective measures must be developed to protect coral spat from fouling.

Artificial surfaces are rapidly colonised in tropical water by a variety of benthic organisms. This begins with the formation of biofilms consisting of biomacromolecules, bacteria, diatoms, and microalgae followed by a shift to macrofouling by macroalgae and invertebrates^38,39^. Reducing this colonisation on ships and marine structures can be achieved with preventative coatings, some of which may be applicable to reduce the colonisation of seeding devices. Conventional antifouling coatings (AFCs) incorporate one or more biocides such as copper, zinc, Irgarol 1051, and diuron^40^. However, these constituents are generally toxic to non-target species such as corals^41–44^ and are unlikely to be approved for use in restoration projects. Nevertheless, copper-containing AFCs have been effective in preventing fouling on structures and trays used in coral farming, though toxicity has been observed when in close proximity to corals^11^. Biocide-free fouling release coatings (FRCs) have been developed as a more environmentally sustainable alternative to AFCs^41^. These coatings, typically hydrophobic or amphiphilic poly (dimethylsiloxane) (PDMS) variants, exhibit very low surface free energies, preventing the adhesion of biofilms and later colonisers, which are readily dislodged by shear forces of water movement^45,46^. In fact, there is evidence of hydrophobic non-toxic wax used in fouling prevention dating back over 2000 years^47^. FRCs and other novel coatings have been successfully used in aquaculture to reduce maintenance costs associated with the manual removal of marine growth^48^; however, their efficacy in preventing colonisation of artificial surfaces on coral reefs has not been tested.

Only two aquarium studies have directly tested the potential of FRC and AFCs to protect coral spat from benthic competition. The first applied non-toxic paraffin wax, with or without 0.1% silicone oil, to terracotta coral settlement tiles^49^. Wax coatings halved the fouling surrounding coral spat (mainly filamentous algae) after 39 d in flow-through mesocosms, and approximately doubled spat survival compared to uncoated surfaces. The second study assessed a range of innovative AFCs and FRCs on ceramic settlement tabs over 37 d in tropical mesocosms^50^. Uncoated tabs developed 96% algal cover which was reduced to 83% cover by an antiadhesive silica based FRC formed a sol–gel method^51^). Adding the slow-release biocide dichlorooctylisothiazolinone (DCOIT) to the sol-gel reduced fouling to 37%. Although considered less toxic to corals than other biocides DCOIT may still affect larval behaviour^50,52^. Modification of the sol-gel coating with cerium dioxide nanoparticles proved ineffective. None of these coatings affected direct coral larvae settlement, indicating low toxicity to corals and could therefore be suitable for application in coral aquaculture for restoration purposes. Although conducted over short periods under artificial conditions, these studies highlight the potential for FRCs and AFCs to protect corals from competition with aggressive early colonisers, an approach worth investigating over longer periods in the field.

This study outlines a proof-of-concept field experiment testing the efficacy of two commercially available non-biocidal FRCs and a wax coating to reduce fouling on coral seeding devices. We also assessed whether the coatings affected survival of attached coral microfragments (as a proxy for coral spat which were unavailable). In this experiment, a shallow mid-shelf reef habitat was seeded with microfragments of the coral *Acropora millepora* on ceramic deployment devices. The ~ 4-cm diameter cores of each device, adjacent to the corals, were treated with one of two FRCs, paraffin wax or were left uncoated (i.e., control); surfaces where fragments were attached were not FRC treated. Fouling, coral survival and surrounding benthic habitat were monitored periodically for 46 weeks at three deployment sites on Davies Reef (Great Barrier Reef, Australia) in 2022. The study aimed to test the efficacy of surface coatings to inhibit competitive algal fouling on device cores and any subsequent effects on coral microfragment survival, as well as the potential influence of benthic habitat on fouling development among surface treatments.

## Methods

### Coating of seeding devices – treatments

**Fig. 1:**
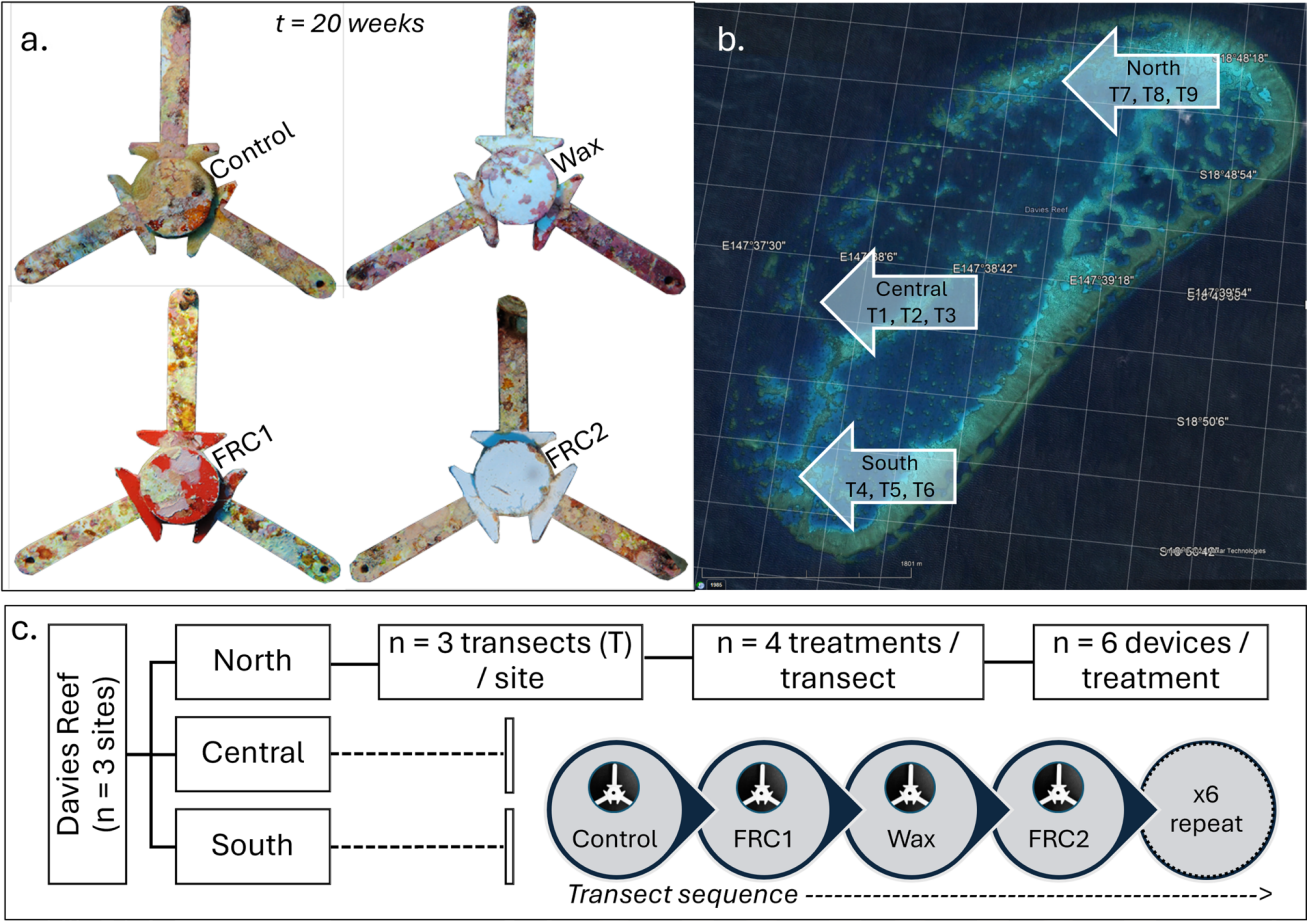
(**a**) Example images of seeding devices at 20 weeks displaying different levels of fouling according to treatment: Control, Wax, FRC1 (red colour), FRC2 (white colour). (**b**) Sites distribution at Davies Reef displaying north, central, and southern deployment areas, Google Maps capture (https://www.google.com.au/maps) (**c**) Schematic of the experimental design across sites, transects and treatments. Transect = sequence of four treatments replicated six times (*n* = 54 devices in total per treatment).

 The seeding devices were star-shaped, with three 5 cm arms (Fig. 1a). Each device featured a ~ 4 cm diameter core with three slots designed to hold 14 × 14 mm concrete settlement tabs, where coral microfragments were attached with glue (see below). The core also included two triangular side protrusions on each side of the slots, designed to limit accidental grazing of the corals by corallivores^29^. The concrete tabs were secured in place by a ceramic spindle. The devices, made from 95% alumina ceramic (i.e., fully sintered aluminum oxide, Al_2_O_3_) were manufactured at Shanghai Gongtao Ceramics CO., Ltd. PRC (www.gongtaoceramics.com) (see detailed specifications in^24^.

A total of 216 seeding devices were used across four treatments: “Control” (uncoated), paraffin “Wax”, “FRC1” and “FRC2” (Fig. 1a; Table 1, Table S2.1), totalling 54 devices per treatment. Coating was applied by hand only to device cores, with the three arms left uncoated to allow for benthic algal colonisation to consolidate devices to the reef substratum (for details see Table S2.1).

The food grade wax (CoralCare) was the same coating applied to coral settlement surfaces by Tebben et al. (2014). The wax was heated to 65 °C and applied to seeding devices with paint brushes (Table 1; Table S2.1). Two commercial FRCs were also applied to seeding devices by brush following manufacturer’s instructions (Table S2.1). Intersleek 1001 (FRC1) is an amphiphilic silicone fluoropolymer foul release coating, incorporating biorenewable long chain waxy sterols from lanolin^53^. Hempasil 77,300 (FRC2) is a hydrophobic silicone foul release coating^54^. Neither product contains biocides, and each complies with the International Convention on the Control of Harmful Antifouling Systems on Ships as adopted by IMO October 2001^55^.

**Table 1:** Coating treatments applied to the seeding devices. See table S2.1 for more details regarding source and application methods.

Treatment	Name	Description
Control	No coating	Bare alumina ceramic
Wax	CoralCare	Food grade waxes^56^.
FRC1 (red)	Intersleek 1001	An amphiphilic silicone fluoropolymer foul release coating, incorporating biorenewable long chain waxy sterols from lanolin (the wax on sheep’s wool)^53^. Red colour.
FRC2 (white)	Hempasil 77,300	A hydrophobic silicone foul release coating^54^. White colour.

### Asexual propagation of coral fragments

Three *Acropora millepora* colonies (up to 25 cm diameter) were collected from Davies Reef (18.82°S, 147.65°E) in February 2022 (GBRMPA permit No. G12/35236.1) and transferred to the National Sea Simulator (SeaSim) at the Australian Institute of Marine Science, Townsville Australia. Colonies were maintained in 1700 L semi-recirculating tanks at ~ 28 °C, which is the same temperature as the collection site. A total of 400 (8 × 8 mm) fragments were generated from the three colonies and were individually glued with cyanoacrylate glue (Gorilla super glue gel) onto each of 400 concrete Table (14 × 14 mm per tab) which together form a larger concrete tile (280 × 280 mm) (Fig S1). Corals were cut into fragments using a wet diamond bandsaw (Gryphon C-40). The resulting tiles were kept under ambient conditions for two weeks to allow for tissue healing and fed daily (2000 cells mL^−1^ of a mix of *Tisochrysis lutea*, *Nannochloropsis oceanica*,* Pavlova lutheri*, *Dunaliella* sp.; 1.5 nauplii mL^−1^ *Artemia salina*). The surrounding concrete area to each fragment was not coated with any treatment (Fig S1).

### Reef deployment

Microfragments from all three colonies were included in every device, with each of three tabs having a single healthy microfragment from one of the colonies. The same sequence of colony representative microfragments was maintained across all replicate devices to allow follow up through time. Just before deployment, devices were assembled into stainless steel rods and placed in 60 L flow-through tanks for transportation to the reef. Each rod had a total of 20 devices with five replicates of each treatment.

Deployment was carried out in April 2022 at three reef sites situated on the western edge of Davies Reef from north to south (Fig. 1b, Table S2.2). Devices were diver-deployed directly onto the reef at 4–6 m depth along three replicate transects per site, in which each of the four FRC treatments were replicated five times (Fig. 1c). Transects were ~ 20 m long and were marked by steel reinforcing bars at the start and end with the corresponding transect label (GBRMPA SAP Approved for permit G21/45348.1). Devices were threaded with a nylon line (2 mm) to facilitate census and retrieval with ~ 85 cm separation between sequentially alternating devices from each treatment. Devices were left in direct contact with the substratum but were not directly fixed, although the loose nylon line was nailed to the substratum every 1.5 m.

### Trait assessment and data processing

**Fig. 2:**
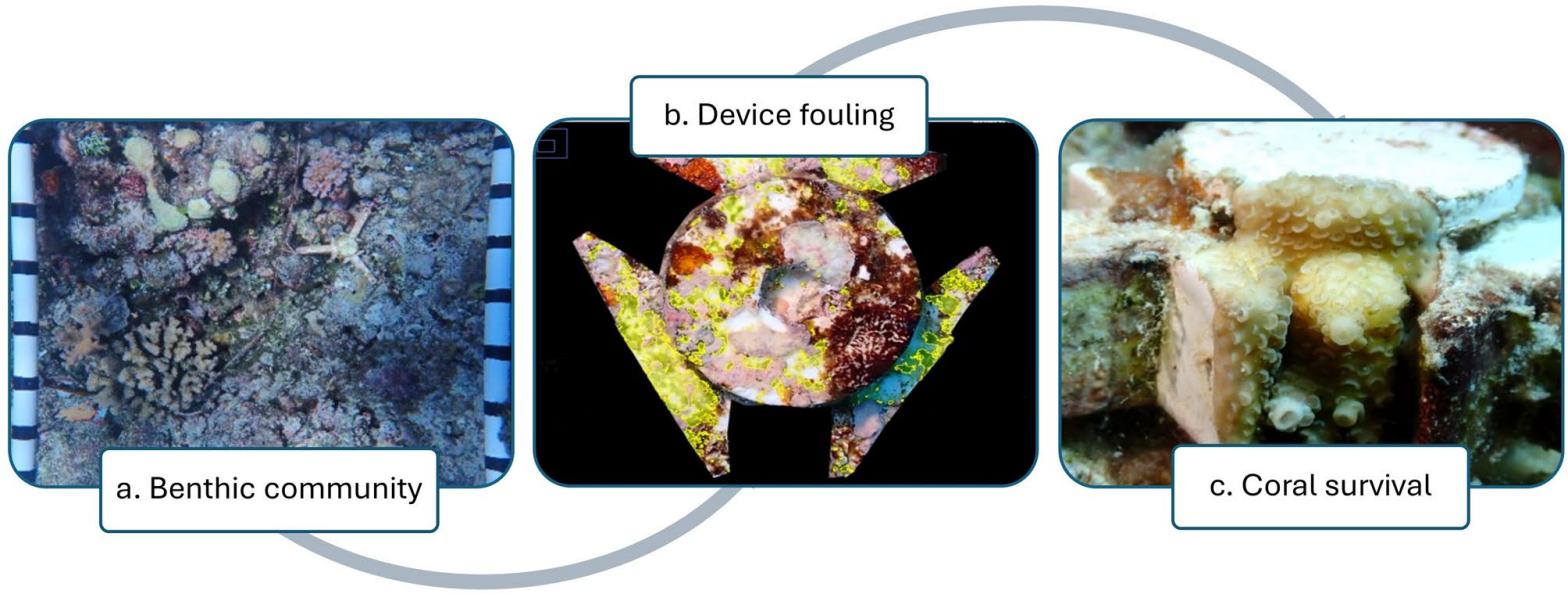
(**a**) Example images obtained from the benthic quadrat, (**b**) device fouling “core” and (**c**) fragment survival on concrete tabs during each censusing timepoint.

 Device census was performed on SCUBA approximately every three months until retrieval at 46 weeks. Census consisted of photography of: (1) individual devices to assess fouling across treatments; (2) individual tabs to assess survival of coral microfragments; and (3) benthic community within 50 × 50 cm quadrats surrounding each device (Fig. 2). The benthic quadrats allowed a performance comparison of the different antifoulant treatments in a range of reef environments as well as their potential influence on the survival of coral fragments and correlations with specific benthic taxa. Images were taken using an Olympus TG-6 camera with Ikelite housing on underwater HDR mode for benthic quadrats and devices, or underwater microscope function for individual tabs. A diving light (SeaLife/SeaDragon 2500 Lm, intensity level 1 [33%]) was mounted on the housing to improve image quality.

### Device fouling

 FRC treatment of seeding device cores is likely to reduce fouling by benthic organisms. Images from the exposed side of the device were assessed with ImageJ^57^. Images were divided into the arms and the core to allow comparisons of fouling patterns between treated and untreated surfaces. The process encompassed the aggregation of pixels with similar colours (such as k-means clustering^58^) using the combination of plug-ins (eyedropper selection + versatile wand) to increase time efficiency, allowing the capture of the colour spectrum (i.e., from light to dark). Colours were used to classify major fouling communities and included pink (CCA, crustose coralline algae), green or brown/red (algae), light grey (sediments or dead CCA within the “other” category) The remaining categories, such as live coral tissue (i.e., used to capture growth from fragments) or “other” invertebrates (e.g., bryozoans, sponges), required manual tracing of the areas of interest to due colour similarities among categories; however, this did not substantially increase time requirements due to their overall low coverage. Each of these fouling groups encompassed a range of marine fouling taxa and their representative colour (Table S2.3) which were consolidated to simplify the analysis. Non-fouled surfaces were classified as “clear” and included original white ceramic and FRC coatings.

Fouling data from the different categories of marine fouling was converted to proportion of area covered on the surface of each device. Total fouling was the proportion of the device covered by CCA, brown, or green algae. Data analyses were performed in R version 4.0.3^59^. To quantify fouling in the device core (i.e. proportion of device covered by fouling), we fitted a series of generalised linear mixed effects models in a Bayesian framework using the ‘brms’ package^60^. A total of eight models were fitted, each with a different combination of response variables: treatment, site, experimental timepoint, and their corresponding two-way interactions (Table S2.4). All models included a random effect of genotype (colony), and a random effect of device nested in transect. Since the response variable was constrained between values 0 to 1 (i.e., proportion of the device core with fouling), a zero-one inflated beta distribution was assumed. A three-way interaction was not included since the model had difficulty converging. Each model had 3 chains, each with 5000 iterations (half of which were discarded during warm-up), and a thinning of 5. The function ﻿“get_prior’’ from the ‘brms’ package was used to obtain weakly informative priors. Models were compared using leave-one-out cross-validation (LOO) with the ‘loo’ package^61^. Additionally, a pairwise comparison was established to compare individual treatments across sites in the best-fit model.

### Survival of coral microfragments

FRC treatment of device cores may indirectly improve the survival of coral microfragments. Images of the individual tabs were scored to quantify survival of coral fragments from each representative colony. The assessment consisted of live versus dead fragments and were categorised as 1 or 0, respectively. To account for potential partial mortality, fragments with less than 5% of healthy live tissue were considered dead at the time of censusing. Some microfragments appeared to be less pigmented than others, but these were considered alive at the time of census. No bleaching of surrounding colonies was observed.

Survival was quantified following the same approach as per device fouling. However, a model including a three-way interaction between treatment, site, and experimental timepoint was also included in the comparison^59,60^ (Table S2.5). Survival models assumed a Bernoulli distribution with a logit link. Each model had four chains with 4000 iterations each (2000 of which were discarded as warm-up) and a thinning of 1. Priors were obtained using the function ‘get_prior’ as described above. Models were compared using LOO validation as described above. In addition, a pairwise comparison was established to compare individual pairs of treatments across sites using the best-fit model.

### Benthic community

 The surrounding benthic composition of seeding devices may change over time, impacting fouling and or coral survival. To characterise the benthic community in each quadrat, quadrat images from all experimental time points were uploaded to ReefCloud^62^which is a machine learning artificial intelligence software for benthic image classification. The label set of categories was a modified version of the ReefCheck (GBRMPA) database^63^ to include additional taxa of interest and scientific equipment (e.g., alumina device) (Table S2.6). A total of 20 randomly allocated points of interest were added to each image, and then 30% of the total annotated points were manually (human) classified against the label set as suggested by ReefCloud procedures for machine training. Images were randomly checked to confirm that the resulting machine classification was accurate with that level of algorithm training.

The resulting annotated dataset was exported and processed using R. A few taxa were clustered into more general categories to allow for a clearer description of benthic communities around the devices and potential correlation analyses (Table S2.7). These categories included percent cover of coral, macroalgae, recently dead coral, rock or rubble covered with either CCA or turf algae, sand, silt, soft coral, and sponges across all sites. Other categories without numeric values were removed from the dataset. Permutational multivariate analysis of variance and principal-component-analysis (PCoA) (based on Bray-Curtis dissimilarity distance) were used to assess differences on the percentage cover of benthic community across sites and experimental timepoints with the R package ‘vegan’^64^.

### Relationship between benthic composition and coral microfragment survival

Coral survival could be influenced by particular benthic taxa. To test whether including data on benthic categories improved the predictive performance of the model, we compared the best-fit model from microfragment survival analysis with eight additional models that included one of four key benthic categories as a fixed effect (turf algae, CCA, sediment, or marine invertebrates other than coral). Each model had the same baseline structure as the best-fit model from microfragment survival, plus one of the benthic categories (as percent cover) either by itself or interacting with treatment (Table S2.8). Only one benthic category was included per model to avoid issues with collinearity. Models were compared using LOO validation and Spearman rank correlation test was performed to understand particular interactions among response variables.

## Results

### Device fouling

**Fig. 3:**
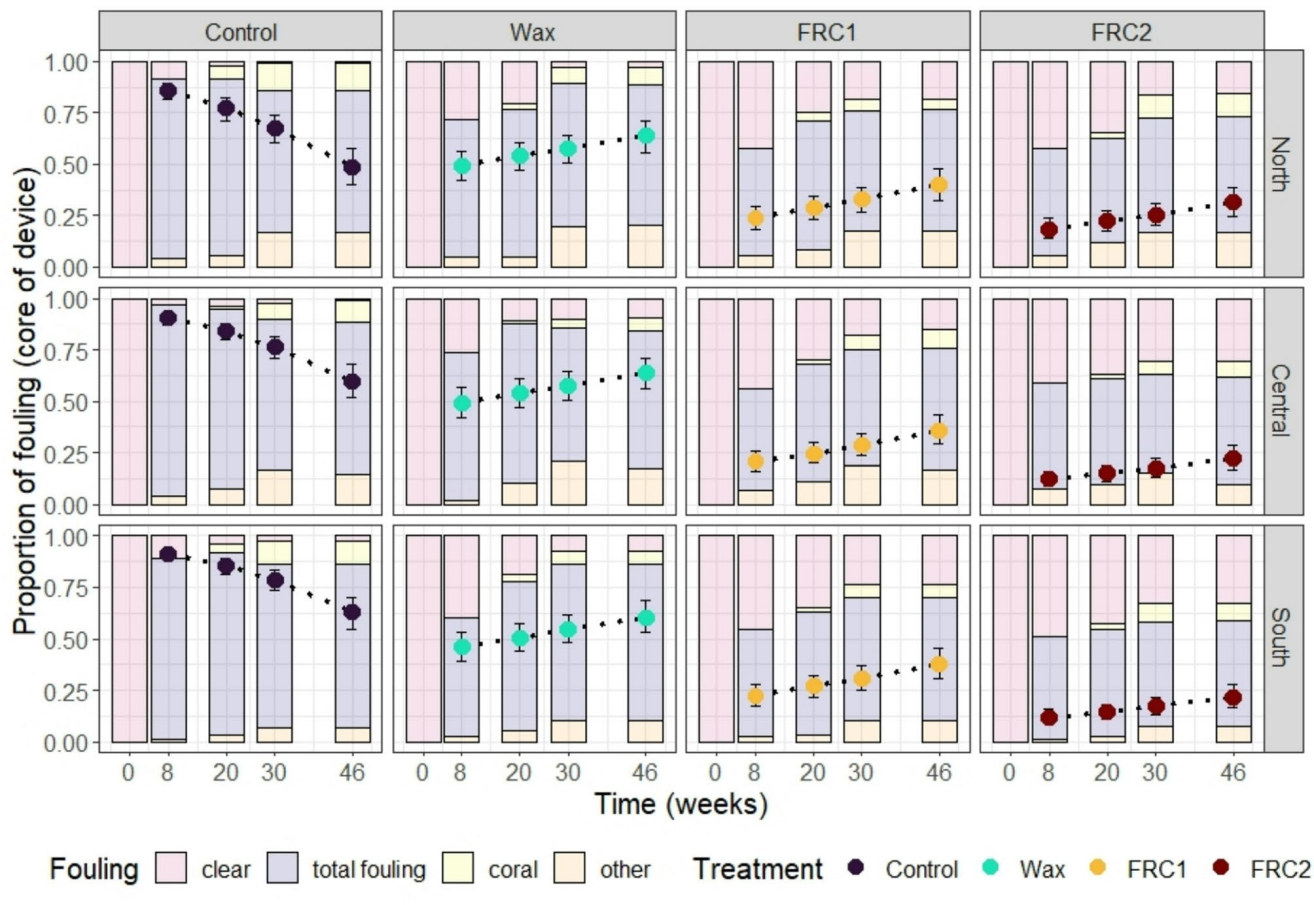
Fouling of devices deployed at three Davies Reef locations. Dots represent fitted model predictions for total fouling including live CCA, green algae and brown/red algae (excluding coral, dead CCA and sediment). Dot colours represent the four different treatments and error bars represent 95% confidence intervals. Bar plots in the background represent the total proportion of fouling categories within each treatment, timepoint and location. Categories are represented by each colour, with “other” incorporating dead CCA and/or associated sediment.

 The device cores became progressively colonised with CCA, green and brown algae (together described as total fouling) over the duration of deployment (Fig. 3, Table S2.9-10). Some of the early colonising CCA died and often retained sediments (classified as *other*), while after 30 and 46 weeks some of the microfragments had grown from the concrete tabs onto the surface of the device cores (classified as *Coral*, Fig. 2c). The apparent reduction in fouling of control devices after eight weeks (Fig. 3) was primarily due to microfragment coral growth and sediment accumulation, which occupied more available surface area thereby reducing the proportion covered by live CCA, green, brown, and red algae. However, clear surface area continued to decline on control devices, falling to less than 3% by 48 weeks—lower than on any of the treated devices. The uncoated arms of all devices became almost completely colonised by CCA, green and brown algae after 8 weeks regardless of the treatment (core coating) (Fig. S2.2). Fouling on the device arms was therefore not quantitatively assessed.

The best-fit model included the interaction of treatment with time plus the interaction of treatment with location, as well as the inclusion of genotype and device nested in transect as random effects (Table S2.4; R^2^ of 0.65, C.I.: 0.63–0.67). Overall, device cores coated with FRC2 had the least fouling across sites and timepoints, followed by FRC1, Wax and then Control (Fig. 3, Table S2.9, Table S2.10). By the end of the 46-week deployment, the mean proportion of clear surfaces (non-fouled) ranged across all sites from 16 to 33% (FRC2), 15 to 24% (FRC1), 2.6 to 9.7% (Wax) and 0.7 to 2.8% (Control). The total fouling was least on FRC2-coated cores (51 to 56%) and greatest on the Control cores (69 to 79%). The core area overgrown by coral averaged 9% (FRC2), 6.5% (FRC1), 7.1% (Wax), and 12% (Control) across sites, whereas the area covered by sediment and dead CCA (*other*) averaged 11% (FRC2), 15% (FRC1), 16% (Wax), and 13% (Control).

The rate of colonisation on device cores varied strongly between treatments. Like the untreated device arms, the cores of untreated Control devices became heavily (89–92%) colonised with CCA, green and brown algae (total fouling) after only 8 weeks (Fig. 3, Fig. S2.1-2). The next most rapid colonisation by algae was on the Wax-coated cores, which exhibited 27–48% total algal cover (Fig. 3), while FRC1 and FRC2-coated cores exhibited between 10 and 20%, and 7–15% total algae at this early time point, respectively. The Controls, FRC1 and FRC2 cores were colonised by diverse algal communities although dominated by brown algae (diatoms), while the Wax-coated cores were colonised primarily by CCA (Fig. 3, Fig S2.1).

### Coral survival

**Fig. 4:**
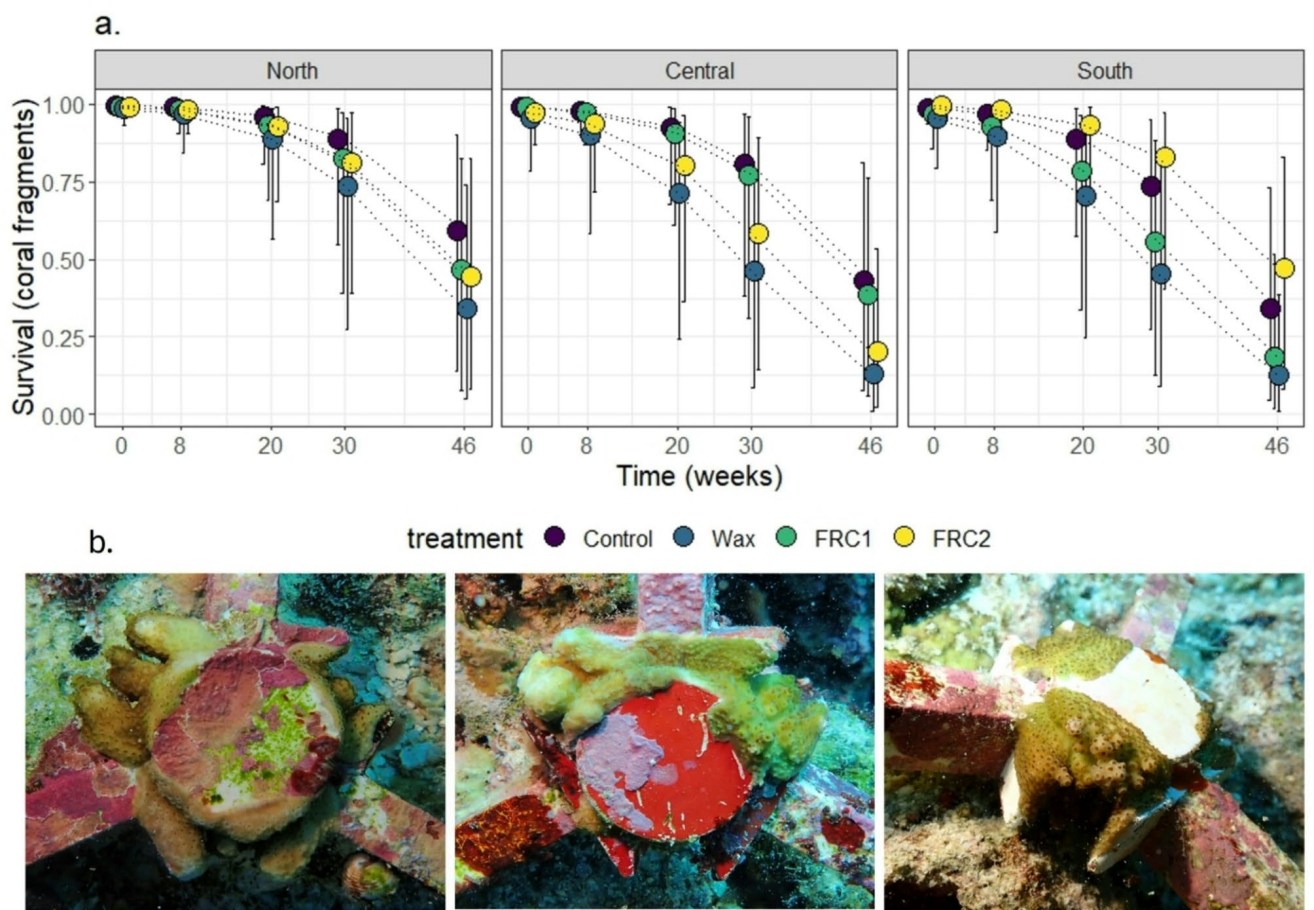
(**a**) Fitted model predictions for survival of coral fragments deployed at three different locations (North, Central, South) at Davies Reef. Colours represent the four different treatments. (**b**) Example images displaying tissue growth on Wax (left), FRC1 (middle) and FRC2 (right) surfaces on seeding devices at 46 weeks.

 The survival of coral microfragments reduced over time to between 13 and 61% after 48 weeks (Fig. 4). The best-fit model included the interaction of core treatment with site and the addition of time as response variables with the inclusion of coral genotype and device nested on transect as random effects (Fig. 4, Table S2.4; R^2^ of 0.55, C.I.: 0.53–0.58). This suggests that the effect of core treatment on survival changes depending on site, but that effect was consistent over time. In addition, pairwise comparisons revealed differences in survival for several combinations of core treatments and sites (Table S2.11). For example, FRC2 devices at southern sites displayed higher survival relative to central sites (which might be attributed to differences on their benthic community, see benthic community Sect. 3.3). While the relative influence of FRC1 and FRC2 coatings on survival appeared different between sites, and the survival on Wax-coated devices was generally the lowest, there was little overall influence of core treatment on coral survival. Furthermore, there was no evident relationship between microfragment survival and the proportion of clear surfaces on each of the core treatments across timepoints (Fig. S2.3).

### Benthic community assessment

**Fig. 5:**
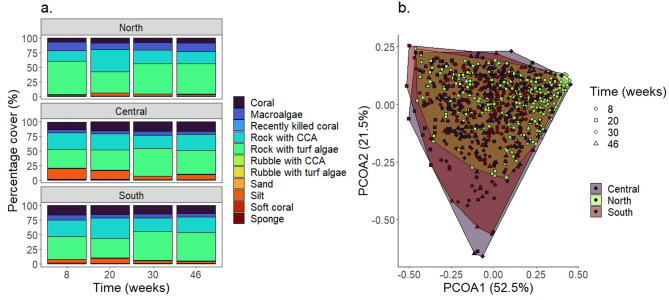
Proportion of clustered benthic categories across sites and experimental timepoints. (**b**) Principal component analysis of benthic community composition highlighting overall similarities among sites.

 Seeding devices can be exposed to different benthic taxa depending on reef locations and time. Benthic community composition varied significantly between sites and transects based on the selected benthic categories (Table S2.12); however, differences were stronger between central and south or central and north sites than between south and north (Table S2.13). In contrast, little variation was observed between benthic communities within each site over the duration of deployment. The most abundant categories across all sites included rock covered with turf algae and rock covered with CCA (Fig. 5, Table S2.14 and S2.15). Moreover, the proportion of coral cover (~ 7%) and rock with CCA (~ 20%) observed on northern sites were half the coverage observed on central sites (18 and 30%, respectively), whereas a similar comparison between central and southern sites did not reveal major differences. Interestingly, macroalgae was similar in abundance at the central (5%) and southern (8%) sites but was higher at the northern sites (15%).

### Correlation of benthic categories and survival of coral fragments

 The best-fit model included the percentage of marine invertebrates (other than coral), without an interaction with treatment (Table S2.8; R^2^ of 0.50, C.I.: 0.46–0.53). Pairwise comparisons across treatments and sites revealed that the majority were not different (i.e., their credible intervals overlapped with zero) when relating survival on devices and invertebrate proportion on their surrounding environment (Table S2.16). In some instances, survival estimates were higher for specific sites and treatment combinations, but that dissimilarity pattern occurred due to distinctions with devices coated with Wax which seemed to have lower survival rates. A similar pattern was observed when contrasting sites and treatments using the second-best fit model that incorporated turf algae as response variable instead of invertebrates (Table S2.17).

**Fig. 6:**
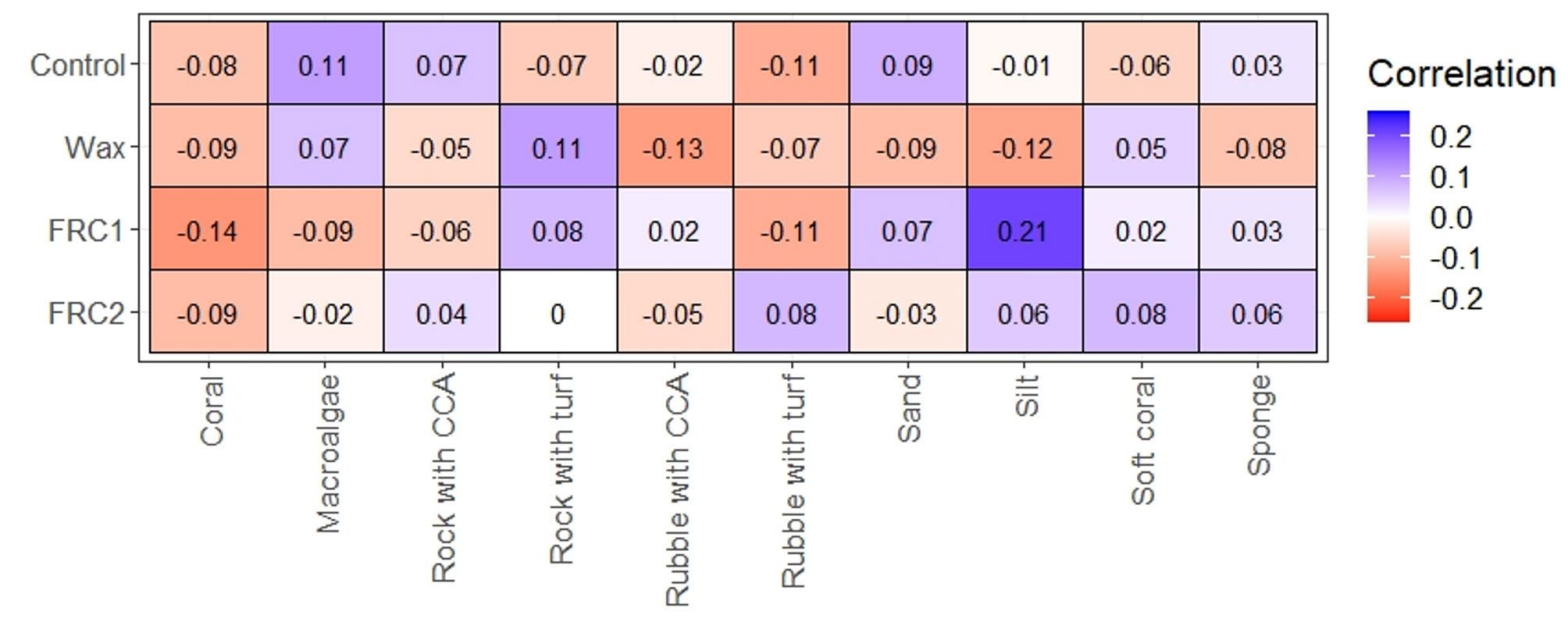
Spearman rank correlation estimates between survival of coral fragment and benthic categories according to treatment. Blue and red colours represent a positive or negative correlation, respectively.

In addition, the Spearman correlation test showed how survival can be influenced by specific combinations of benthic communities around the device and the antifouling treatment applied to its core (Fig. 6). Although these correlations are mostly weak, some patterns provided information on the performance of the treatments. For example, survival of fragments can be expected to be enhanced by the combination of FRC1 on areas with higher silt coverage but reduced on areas with higher coral cover. Interestingly, most benthic categories with FRC2 had a subtle positive correlation relative to other fouling release treatments, which can translate to enhancement of survival of coral fragments.

## Discussion

This study demonstrates that non-biocidal FRCs can significantly reduce early-stage algal colonisation on coral seeding devices, offering a practical solution to one of the major challenges in large-scale coral restoration. The FRC-treated devices maintained substantially more clear surface area than uncoated controls during the critical early weeks post-deployment, when deployed corals are most vulnerable to overgrowth. Importantly, the best-performing coating (FRC2) provided sustained fouling protection over 46 weeks without negatively affecting coral survival, and corals were observed to overgrow the coatings by the trial’s end. These findings highlight the potential of FRCs to enhance the survival of smaller coral spat by reducing early competitive pressure, particularly in high-fouling environments, and support their further evaluation and refinement for use in reef restoration, coral nurseries, and aquaculture systems.

### Influence of FRC coatings on fouling

 The uncoated devices (arms and cores) became rapidly colonised with macroalgae, consistent with previous reports on devices deployed on coral reefs^23,65,66^. All FRC treatments provided substantial protection for the device cores against fouling over the first 8 weeks, with FRC1, FRC2 and Wax treatments showing 75%, 83% and 46% –less fouling, respectively, compared with Controls. Early fouling may influence coral seeding success in different ways: while rapid fouling of the device arms could aid in device retention (though not measured), fouling on the device core presents a risk of overgrowth and competition to deployed corals. Our results align with previous studies, such as a mesocosm experiment where paraffin wax reduced fouling by 50% over 39 days^49^. Additionally, FRC1 and FRC2 outperformed a silicone antiadhesive FRC and were comparable to a DCOIT-containing AFC in reducing fouling on surfaces in an aquarium over 37 days^50^. The strong suppression of algal growth on all coated surfaces during the initial 8 to 20 weeks did not positively or negatively affect microfragment survival, but is likely more beneficial for enhancing coral spat survival, as this early post-settlement stage represents their most vulnerable period^27,67,68^. FRC2 and FRC1 continued to significantly inhibit fouling until the end of the 46-week deployment, with on average 54% and 32% – times less fouling on FRC2, FRC1, respectively compared with Controls. This is likely to reduce competition until spat reach the size-escape threshold of approximately 10 mm diameter (typically 3 to 9 months), beyond which mortality substantially declines^25,68^.

The effectiveness of the coatings in reducing fouling is likely attributable to both their composition and durability over the deployment period. FRC1 and FRC2 treatments remained largely intact over the 46-week period, with only minor abrasive scratches from friction or sediment erosion, and possible grazing, which appeared to be rare. In contrast, the wax coatings thinned over time, and after approximately three months, some crystallization and shrinkage of the wax occurred, leading to the formation of fine cracks. The extent of this fracturing was difficult to quantify due to the lack of contrast between treated and untreated areas and was therefore not measured. These cracks exposed the alumina ceramic of the device, which quickly became colonized. By 48 weeks, the total fouling and its composition resembled that of the Control devices. However, the protection provided by the Wax-coated device cores during the initial ~ 20 week field deployement may still enhance spat survival, as demonstrated in aquarium experiments over 39 days using a similar wax^49^.

After eight weeks the predominant fouling on Control devices was CCA followed by green and brown algae and this pattern was similar for the Wax-coated devices, while the FRC1 and FRC2 devices featured lower proportions of CCA. By the end of deployment, brown algae became more dominant across all treatments and the lowest proportion of CCA was on FRC2 followed by FRC1 devices. Successional fouling on coral reefs is expected and dominance will change with physicochemical conditions, seasonally and under the influence of grazers^69^all of which may have contributed to the observed patterns, including the differences in taxa and extent of fouling on device cores between sites. However, the FRC treatments (including Wax) did influence fouling types at the high taxonomic level assessed. This may be attributed to variations in the responses of developing biofilms, recruitment of algal propagules, or the growth of established benthic algae to the surface properties of the coatings^41^. The substantial reduction in algal growth on FRC-coated surfaces is likely to reduce overall competition; however, since both CCA and brown algae are known to compete with and sometimes overgrow smaller coral spat^70–72^it is uncertain whether the shifts in fouling community structure influenced by different coatings would improve coral survival.

### Mechanisms and potential ecological risk of FRCs

 This study aimed to evaluate the effectiveness of various non-biocidal treatments in reducing aggressive fouling on coral seeding devices deployed directly onto coral reef substratum. All FRCs were assumed to act via physical rather than chemical mechanisms^41^. FRC1 is a fluoropolymer modified with natural lanolin (wool wax), which is designed to enhance hydrophobicity and reduce microfouling, while FRC2 is a hydrophobic silicone fouling release coating (Table 1). Both coatings reduce surface tension and adhesive strength, allowing water movement to dislodge weakly attached organisms^41^. The hydrophobic Wax similarly reduces adhesive strength, proving effective in reducing fouling by algae and invertebrates^73,74^. FRCs typically perform best on fast-moving vessels^75^but water velocity at Davies Reef (likely less than one knot^76^) was sufficient to clear poorly attached algae. Efficacy may be even higher at more exposed reef sites though this remains to be untested.

The commercial FRCs are biocide-free, and the paraffin wax is food-grade, suggesting minimal toxicity. However, the advantages of reducing fouling on the seeding devices with FRCs should be weighed against any potential environment risks. While FRC1 and FRC2 contain modified silicones, their exact formulations are unknown, complicating risk assessments. Silicone does not dissolve or bioaccumulate in marine environments and is reported to have low toxicity to aquatic organisms^77^. However, silicones can persist and may attach to sediments, potentially affecting organisms at high concentrations. This is unlikely to be problematic in the case of coral seeding devices, given the slow release from the coatings^78^ and limited coated surface areas. Over time the wax was observed to crystallise, potentially forming microparticles as the amorphous phase degrades faster than the crystalline phase^56^. Although considered safe for human consumption^79^only two studies have assessed marine hazards of wax microparticles^49,79^. These found limited harmful effects on filter-feeding bivalves^80^ and inconsistent sublethal effects in polychaetes^81^. The present study was not designed assess toxicity, but no reduction coral microfragment survival was observed on any FRC treatment. Indeed, coral was observed to grow from the concrete settlement tab across all FRC surfaces, and in most cases was directly attached (Fig. 4b). Growth on FRC2 matched Control levels, while growth over FRC1 and Wax was lower, but no tissue damage or negative effects on were observed. Nor was there a *halo* of negative influence on the fouling organisms on the arms of the devices immediately adjacent to the coatings. These findings, combined with the coatings’ benign composition, suggests limited harmful effects. However, scaling up restoration with FRC-treated devices will require rigorous environmental impact assessments.

### Coral survival

 The survival estimates of *A. millepora* microfragments on Control devices ranged from 34 to 59% across all sites after 46 weeks, similar to previous reports across various species and morphologies^82,83^. Survival was affected by treatment, location and time although genotype and device also contributed to this variation. The random effect of genotype and device had a standard deviation of 1.12 and 2.58, respectively, indicating that more variation in survival is associated with device than with genotype. FRC treatment effects varied across sites; for example, corals on FRC2-coated devices showed moderate survival at the Northern and Central sites but the highest survival at the Southern site. Greater variation in benthic communities was observed between sites rather than within sites. Although algae were the dominant colonizers of device cores, they constituted only 5% (Central) to 15% (North and South) of the immediate habitats. However, while some site-treatment combinations appeared to correspond with higher coral survival, correlations between survival and both benthic composition and antifouling treatments were weak and inconsistent. Notably, FRC2 showed a slight positive effect on survival compared to other treatments, but this trend was not consistent across sites, indicating that any apparent benefits of FRC coatings on survival may be context-dependent and require further investigation. The potential benefits of FRC application on coral survival may also become clearer when tested across a much broader range of habitats, in particular degraded coral reef habitats that are now dominated by macroalgae.

Previous studies have shown much higher survival rates for microfragments compared to spat when deployed on the same devices^29^indicating that microfragments may not serve as ideal proxies for earlier life stages. The microfragments employed in this study (~ 64 mm^2^) were substantially larger than newly settled spat (~ 1 mm^2^), and similar in size to the reported *escape threshold* for coral recruits, above which early mortality declines^25^. This comparatively larger size may account for the absence of a clear relationship between survival and the proportion of clear surfaces on each core treatment. FRC treatments on the other hand are likely to offer stronger protection for smaller spat. For instance, Tebben et al. (2014) reported FRC wax coatings, similar to the Wax treatment in this study, doubled the coral spat survival in aquaria over 39 days. In that experiment, spat settled within millimetres of the wax coating, likely gaining increased protection from benthic competitors. To maximize protection during future field deployments of coral spat seeding devices, it may be advantageous to extend coating application beyond the core to include the concrete settlement tab.

### Future directions and conclusion

 High mortality rates are common among newly settled corals, so improving coral survival on seeding devices is likely to substantially enhance the feasibility and success of large-scale coral seeding programs^5^. This in situ study provides proof of concept that non-biocidal FRC coatings can effectively reduce potentially competitive algal fouling on seeding devices used for coral reef restoration. However, further studies are required to validate this approach, including testing the efficacy of similar coatings to improve spat survival across a wide range of deployment habitats. In this study, the coatings effectively reduced colonisation by algae on device cores at a typical, healthy, mid-shelf reef. Testing the coatings at locations with higher densities of macroalgae, which can hinder coral recruitment^37^and sites with fast-growing benthic invertebrates like bryozoans which can also outcompete coral recruits^84^ would provide additional understanding of their effectiveness.

Further refinement of this strategy is also required to minimize potential environmental risks and reduce costs. Each device core required approximately 1.6 mL of the commercial FRCs, including their tie-coats. At a cost of around $US60 per litre, this amounts to $US0.10 per device—an increase of 10% assuming a base device cost of $US1 each. The Wax coating could be up to 10-fold less expensive. Efficiencies could be achieved by identifying the minimum surface area necessary to protect coral spat effectively (potentially habitat-dependent, with some sites not requiring coatings). The cost of physically applying FRC coatings and wax also needs to be factored in, but this is beyond the scope of the current study. Other sustainable coatings should also be explored and assessed for their impacts on coral and reef organisms. Since fouling protection may only be necessary during the first few months post-deployment, alternative coatings that degrade safely over time may offer an ideal solution. Overall, non-biocidal FRC coatings show promise in reducing competitive fouling on coral seeding devices, though further research is needed to optimize their efficacy, cost, and environmental compatibility across diverse reef habitats. Non-biocidal FRC coatings may also prove beneficial to reef restoration programs by reducing maintenance in ocean nurseries and coral aquaculture facilities.

## Electronic supplementary material

Below is the link to the electronic supplementary material.

Supplementary Material 1

Supplementary Material 2

Supplementary Material 3

## Data Availability

All data generated or analysed during this study are included as supplementary information files.
