# Supplementary material for: Fouling release coatings reduce colonisation of coral seeding devices
Source: Sci Rep. 2025 Jul 5;15:24023. doi: 10.1038/s41598-025-08268-9 (PMC12228731; doi:10.1038/s41598-025-08268-9)
Supplement: Supplementary file 3 — Supplementary Material 3 [file 41598_2025_8268_MOESM3_ESM.docx]

**Fouling release coatings reduce colonisation of coral seeding devices**

**Jose Montalvo-Proano^1,*^, Florita Flores^1^, Andrea Severati^1^ , and Andrew P. Negri^1^**

^1^Australian Institute of Marine Science, PMB No.3, Townsville, QLD 4810, Australia.

[*j.montalvoproano@aims.gov.au](mailto:*j.montalvoproano@aims.gov.au)

**Supplementary material**

**Table S2.1** Coating treatments applied to the seeding devices deployed at Davies Reef, April 2022. *See manufacturer’s website for detailed application method.

| Treatment | Commercial name, description | Application method* | Source, manufacturer |
| --- | --- | --- | --- |
| Control | No coating | NA | NA |
| Wax | CoralCare, a refined food grade paraffin wax | Wax was heated to 65°C and applied to untreated ceramic device foam paintbrush. The coating was approximately 1-1.5 mm thick. | Aqua Firma Services [www.aquafirma.com.au](https://aus01.safelinks.protection.outlook.com/?url=http%3A%2F%2Fwww.aquafirma.com.au%2F&data=05%7C01%7CJ.MontalvoProano%40aims.gov.au%7Cefb8a2897f3e46fbcb0708db4b88b38a%7Ce054a73b40dc4ae39fce60c537aa6fac%7C0%7C0%7C638186822734782836%7CUnknown%7CTWFpbGZsb3d8eyJWIjoiMC4wLjAwMDAiLCJQIjoiV2luMzIiLCJBTiI6Ik1haWwiLCJXVCI6Mn0%3D%7C3000%7C%7C%7C&sdata=lDuw45YZHLooVKvL%2BtWCRZ6Uqt6Dr8D%2BW341awFmMzM%3D&reserved=0) |
| FRC1 (red) | Intersleek 1001 | Applied by foam paintbrush  Coat 1: Intergard 263  Coat 2: Intersleek 737  Coat 3: Intersleek 1001 | AkzoNobel Pty Ltd International Marine <https://www.international-marine.com/en/products/filters/t_Foul-Release-Coatings> |
| FRC2 (white) | Hempasil 77300 | Applied by foam paintbrush  Coat 1: Hempasil Nexus X-Tend 27500  Coat 2: Hempasil 77300 | Hempel (Wattyl) Australia Pty Ltd <https://www.hempel.com/products/hempasil-77300-77300> |

**Figure S1.** Example image of coral microfragments glued into a large flat concrete tile prior to splitting it onto individual small tiles for their addition to ceramic seeding devices (left). Ceramic device displaying coral fragment on settlement concrete tab prior to reef deployment (right). NOTE: a large concrete tile is used to glue replicate fragments until the individual tabs that form the tile are split and then loaded onto each device.

**
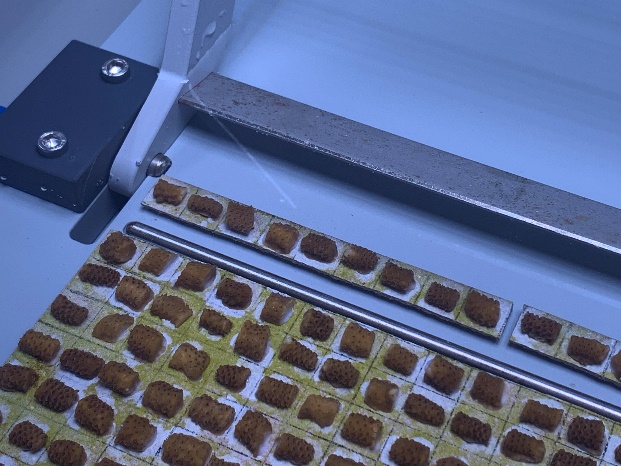
** **
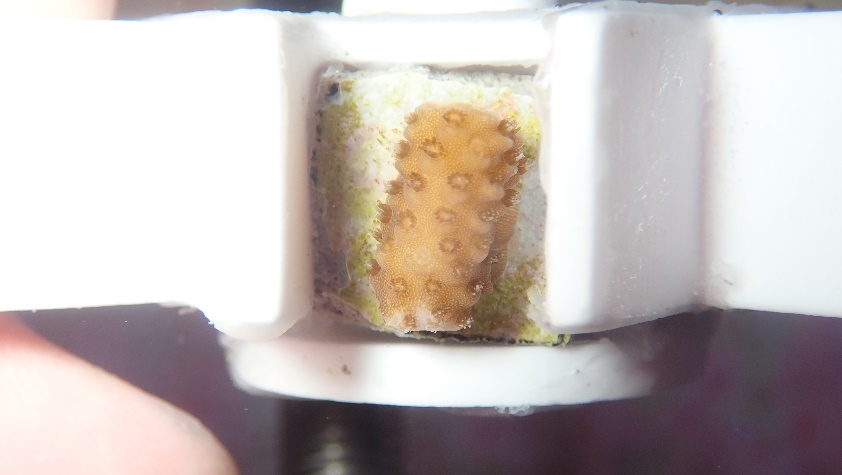
**

**Table S2.2** GPS coordinates for each of the deployment sites on Davies Reef.

| Site: Central = transects 1, 2 and 3 | 18.8291°S, 147.6299°E |
| --- | --- |
| Site: South = transects 4, 5, and 6 | 18.8423°S, 147.6282°E |
| Site: North = transects 7, 8 and 9 | 18.8050°S, 147.6492°E |

**Table S2.3.** Key classification of fouling categories to simplify patterns.

| Fouling category | Representative taxa | Overall fouling category | Colour allocation (Fig. 3 and S2.2) |
| --- | --- | --- | --- |
| Clear | Original substratum (coated, non-coated) – experimental Controls | Clear | 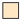 |
| CCA | *Titanoderma* spp*.*, *Mesophyllum* spp*.*, others | Total fouling (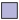) | 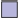 |
| Green algae | Cyanobacteria, filamentous, others |  | 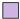 |
| Brown algae | Diatoms, hydroids, others. **Red algae (mostly filamentous) also included. |  | 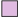 |
| Other | Sediments and dead CCA | Other | 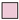 |
| Coral | Coral growing across the core surface of the seeding device | Coral | 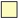 |

**Table S2.4** Statistical models tested to determine the best fit for estimates of device fouling according to core treatment, site, and time. Shaded row displays best fitted model with the lowest looic value.

| Model | Interaction (*) and/or addition (+) | Random effects | Family | LooIC value |
| --- | --- | --- | --- | --- |
| 1 | treatment * time * location *(did not converge, not used)* | colony + device nested in transect | Zero-one-inflated-beta | na |
| 2 | treatment * time + treatment * location + time * location | colony + device nested in transect | Zero-one-inflated-beta | -826.6 |
| 3 (BEST) | treatment * time + treatment * location | colony + device nested in transect | Zero-one-inflated-beta | -827.8 |
| 4 | treatment * time + time * location | colony + device nested in transect | Zero-one-inflated-beta | -810.4 |
| 5 | treatment * location + time * location | colony + device nested in transect | Zero-one-inflated-beta | -662.4 |
| 6 | treatment * time + location | colony + device nested in transect | Zero-one-inflated-beta | -812.1 |
| 7 | treatment * location + time | colony + device nested in transect | Zero-one-inflated-beta | -665.7 |
| 8 | treatment + location * time | colony + device nested in transect | Zero-one-inflated-beta | -656.8 |
| 9 | treatment + location + time | colony + device nested in transect | Zero-one-inflated-beta | -658.0 |

**Table S2.5** Statistical models tested to determine the best fit for estimates of coral survival according to core treatment, site, and time. Shaded row displays best fitted model with the lowest looic value.

| Model | Interaction (*) and/or addition (+) | Random effects | Family | LooIC value |
| --- | --- | --- | --- | --- |
| 1 | treatment * time * location | colony + device nested in transect | Binomial | 2201.8 |
| 2 | treatment * time + treatment * location + time * location | colony + device nested in transect | Binomial | 2197.7 |
| 3 | treatment * time + treatment * location | colony + device nested in transect | Binomial | 2199.7 |
| 4 | treatment * time + time * location | colony + device nested in transect | Binomial | 2195.7 |
| 5 | treatment * location + time * location | colony + device nested in transect | Binomial | 2197.1 |
| 6 | treatment * time + location | colony + device nested in transect | Binomial | 2203.8 |
| 7 (BEST) | treatment * location + time | colony + device nested in transect | Binomial | 2193.1 |
| 8 | treatment + location * time | colony + device nested in transect | Binomial | 2202.0 |
| 9 | treatment + location + time | colony + device nested in transect | Binomial | 2198.8 |

**Table S2.6** Label set used for ReefCloud categorisation of benthic community. Label set is based on the ReefCheck database with a few inclusions to target taxa of interest.

| CODE | DESCRIPTION | FUNCTIONAL GROUP |
| --- | --- | --- |
| OT | All non-target life forms | Other |
| SC | All other soft coral | Soft Coral |
| SP | All other sponges | Sponge |
| DEVICE | Alumina device | Other |
| INV-Anem | Anenome | Invertebrate Indicator |
| OT-Ascid | Ascidians | Other |
| MA-Asp | Asparagopsis | Macroalgae |
| HCB | Bleached hard coral | Hard Coral |
| SCB | Bleached soft coral | Soft Coral |
| HCBR | Branching hard coral | Hard Coral |
| INV-Bryazoan | Bryazoan | Invertebrate Indicator |
| NIA-Cau | Caulerpa | Nutrient Indicator Algae |
| NIA-Chl | Chlorodesmis | Nutrient Indicator Algae |
| SD | Coarse grain particulate matter | Sand |
| INV-UrColl | Collector urchin | Invertebrate Indicator |
| Background | Cropped background | Other |
| INV-COTS | Crown-of-thorns starfish | Invertebrate Indicator |
| HCE | Encrusting hard coral | Hard Coral |
| SPE | Encrusting sponge | Sponge |
| MA-EuchKappa | Eucheuma/Kappaphycus | Macroalgae |
| SI | Fine particulate matter | Silt |
| HCF | Foliose hard coral | Hard Coral |
| INV-Clam | Giant clam | Invertebrate Indicator |
| MA-Hal | Halimeda | Macroalgae |
| SCL | Leathery soft coral | Soft Coral |
| NIA-Lob | Lobophora | Nutrient Indicator Algae |
| INV-UrDia | Long-spined black sea urchin | Invertebrate Indicator |
| MA | Macroalgae | Macroalgae |
| HCM | Massive hard coral | Hard Coral |
| NIA | Most other algae forms | Nutrient Indicator Algae |
| HC | Other growth forms | Hard Coral |
| MA-Pad | Padina | Macroalgae |
| INV-UrPen | Pencil urchin | Invertebrate Indicator |
| HCP | Plate hard coral | Hard Coral |
| RKC | Recently killed coral (not covered with algae) | Recently Killed Coral |
| NIA-RedAlgae | Red algae | Nutrient Indicator Algae |
| RKCNIA | RKC covered with nutrient indicator algae | Recently Killed Coral |
| RKCTA | RKC covered with turf algae | Recently Killed Coral |
| RC | Rock (not covered with algae) | Rock |
| RCCA | Rock covered with coralline algae | Rock |
| RCTA | Rock covered with turf algae | Rock |
| RB-cca | Rubble covered with CCA | Rubble |
| RB | Rubble covered with turf | Rubble |
| MA-Sar | Sargassum | Macroalgae |
| INV-SeaC | Sea cucumber | Invertebrate Indicator |
| TAPE | Transect tape or other scientific equipment | Other |
| MA-Tur | Turbinaria | Macroalgae |
| UNK | Unknown or unclear | Other |
| SCZ | Zoanthids | Soft Coral |

**Table S2.7** Key classification of benthic categories used in the analysis of quadrat images across sites.

| Group | Benthic taxa included |
| --- | --- |
| Coral | Other (hard coral growth forms), bleached hard coral, braching hard coral, encrusting hard coral, foliose hard coral, massive hard coral, plate hard coral |
| Invertebrate | Anenome, giant clam, crown-of-thorns starfish, sea cucumber, collector urchin, long spined black urchin, pencil urchin, ascidians |
| Macroalgae | Asparagopsis, Halimeda, Padina, Sargassum, Turbinaria, most other algae forms, Caulerpa, Chlorodesmis, Lobophora, recently killed coral covered with nutrient indicator algae, recently killed coral covered with turf algae, other macroalgae |
| Recently killed coral not covered with algae | Recently killed coral not covered with algae |
| Other | Cropped background, alumina device, all non-target life forms, transect tape or scientific equipment, unknown or unclear |
| Soft coral | All other soft coral, bleached soft coral, leathery soft coral, zoanthids |
| Sponges | All other sponges, encrusting sponge |

**Table S2.8** Statistical models tested to determine the best-fit for estimates potential relationship between the benthic categories with the survival of microfragments according to core treatment and across different timepoints. Shaded row displays best fitted model with the lowest looic value.

| Model | Interaction (*) and/or addition (+) | Random effects | Family | LooIC value |
| --- | --- | --- | --- | --- |
| 1a | treatment * location + time + treatment * turf_macro | device nested in transect | Binomial | 1727.0 |
| 1b | treatment * location + time + turf_macro | device nested in transect | Binomial | 1718.2 |
| 2a | treatment * location + time + treatment * CCA | device nested in transect | Binomial | 1721.6 |
| 2b | treatment * location + time + CCA | device nested in transect | Binomial | 1722.2 |
| 3a | treatment * location + time + treatment * sediment | device nested in transect | Binomial | 1719.0 |
| 3b | treatment * location + time + sediment | device nested in transect | Binomial | 1720.1 |
| 4a | treatment * location + time + treatment * invertebrate | device nested in transect | Binomial | 1720.7 |
| 4b (BEST) | treatment * location + time + invertebrate | device nested in transect | Binomial | 1718.0 |
| 5 | treatment * location + time | device nested in transect | Binomial | 1723.5 |

**Table S2.9** Pairwise comparison of fouling of device (core) estimates (mod#3) according to treatment and location. Light grey rows showing the 1^st^ group having higher fouling than the 2^nd^, dark grey showing 1^st^ group having lower fouling than the 2^nd^, and “na” showing no difference between groups.

| Contrast (1^st^ vs 2^nd^) | estimate | lower.HPD | upper.HPD | difference |
| --- | --- | --- | --- | --- |
| Control central - FRC2 central | 3.063106 | 2.765365 | 3.371193 | 1^st^ > 2^nd^ |
| Control central - FRC1 central | 2.424638 | 2.148944 | 2.720202 | 1^st^ > 2^nd^ |
| Control central - Wax central | 1.163492 | 0.882461 | 1.446907 | 1^st^ > 2^nd^ |
| Control central - Control north | 0.448136 | 0.040058 | 0.857173 | 1^st^ > 2^nd^ |
| Control central - FRC2 north | 2.576441 | 2.158494 | 2.978796 | 1^st^ > 2^nd^ |
| Control central - FRC1 north | 2.229971 | 1.843108 | 2.649715 | 1^st^ > 2^nd^ |
| Control central - Wax north | 1.169437 | 0.769887 | 1.575916 | 1^st^ > 2^nd^ |
| Control central - Control south | -0.11547 | -0.5331 | 0.291144 | na |
| Control central - FRC2 south | 3.106057 | 2.670494 | 3.508787 | 1^st^ > 2^nd^ |
| Control central - FRC1 south | 2.319982 | 1.902368 | 2.71705 | 1^st^ > 2^nd^ |
| Control central - Wax south | 1.30899 | 0.897811 | 1.66302 | 1^st^ > 2^nd^ |
| FRC2 central - FRC1 central | -0.63613 | -0.91595 | -0.3345 | 1^st^ < 2^nd^ |
| FRC2 central - Wax central | -1.89865 | -2.21429 | -1.63251 | 1^st^ < 2^nd^ |
| FRC2 central - Control north | -2.61483 | -3.05104 | -2.20906 | 1^st^ < 2^nd^ |
| FRC2 central - FRC2 north | -0.48096 | -0.89644 | -0.06382 | 1^st^ < 2^nd^ |
| FRC2 central - FRC1 north | -0.83072 | -1.22158 | -0.42127 | 1^st^ < 2^nd^ |
| FRC2 central - Wax north | -1.90371 | -2.27816 | -1.46121 | 1^st^ < 2^nd^ |
| FRC2 central - Control south | -3.18444 | -3.58408 | -2.73307 | 1^st^ < 2^nd^ |
| FRC2 central - FRC2 south | 0.041825 | -0.40392 | 0.436418 | na |
| FRC2 central - FRC1 south | -0.74236 | -1.15145 | -0.35129 | 1^st^ < 2^nd^ |
| FRC2 central - Wax south | -1.76339 | -2.18427 | -1.36603 | 1^st^ < 2^nd^ |
| FRC1 central - Wax central | -1.25538 | -1.52021 | -0.98849 | 1^st^ < 2^nd^ |
| FRC1 central - Control north | -1.97709 | -2.39659 | -1.59933 | 1^st^ < 2^nd^ |
| FRC1 central - FRC2 north | 0.160477 | -0.24237 | 0.524097 | na |
| FRC1 central - FRC1 north | -0.19452 | -0.56079 | 0.198383 | na |
| FRC1 central - Wax north | -1.26168 | -1.67327 | -0.88977 | 1^st^ < 2^nd^ |
| FRC1 central - Control south | -2.54726 | -2.92762 | -2.14354 | 1^st^ < 2^nd^ |
| FRC1 central - FRC2 south | 0.682896 | 0.306582 | 1.086673 | 1^st^ > 2^nd^ |
| FRC1 central - FRC1 south | -0.10367 | -0.48921 | 0.276873 | na |
| FRC1 central - Wax south | -1.11639 | -1.49928 | -0.75636 | 1^st^ < 2^nd^ |
| Wax central - Control north | -0.71143 | -1.13101 | -0.33302 | 1^st^ < 2^nd^ |
| Wax central - FRC2 north | 1.421962 | 1.014535 | 1.810415 | 1^st^ > 2^nd^ |
| Wax central - FRC1 north | 1.070801 | 0.691589 | 1.465105 | 1^st^ > 2^nd^ |
| Wax central - Wax north | 0.000861 | -0.40138 | 0.381258 | na |
| Wax central - Control south | -1.28534 | -1.68855 | -0.90218 | 1^st^ < 2^nd^ |
| Wax central - FRC2 south | 1.93881 | 1.539046 | 2.364585 | 1^st^ < 2^nd^ |
| Wax central - FRC1 south | 1.14736 | 0.729098 | 1.533852 | 1^st^ > 2^nd^ |
| Wax central - Wax south | 0.137595 | -0.2561 | 0.519655 | na |
| Control north - FRC2 north | 2.136476 | 1.853462 | 2.44163 | 1^st^ > 2^nd^ |
| Control north - FRC1 north | 1.790647 | 1.471853 | 2.061209 | 1^st^ > 2^nd^ |
| Control north - Wax north | 0.709604 | 0.438322 | 1.018328 | 1^st^ > 2^nd^ |
| Control north - Control south | -0.56601 | -0.99104 | -0.18232 | 1^st^ < 2^nd^ |
| Control north - FRC2 south | 2.654921 | 2.27427 | 3.088501 | 1^st^ > 2^nd^ |
| Control north - FRC1 south | 1.8713 | 1.440758 | 2.26427 | 1^st^ > 2^nd^ |
| Control north - Wax south | 0.856827 | 0.46655 | 1.260517 | 1^st^ > 2^nd^ |
| FRC2 north - FRC1 north | -0.35274 | -0.63957 | -0.07687 | 1^st^ < 2^nd^ |
| FRC2 north - Wax north | -1.41821 | -1.70414 | -1.1553 | 1^st^ < 2^nd^ |
| FRC2 north - Control south | -2.70623 | -3.08692 | -2.28267 | 1^st^ < 2^nd^ |
| FRC2 north - FRC2 south | 0.520352 | 0.137321 | 0.936313 | 1^st^ > 2^nd^ |
| FRC2 north - FRC1 south | -0.26626 | -0.65948 | 0.113148 | na |
| FRC2 north - Wax south | -1.27778 | -1.63879 | -0.89102 | 1^st^ < 2^nd^ |
| FRC1 north - Wax north | -1.06845 | -1.35647 | -0.82769 | 1^st^ < 2^nd^ |
| FRC1 north - Control south | -2.35791 | -2.75603 | -1.98425 | 1^st^ < 2^nd^ |
| FRC1 north - FRC2 south | 0.869185 | 0.456761 | 1.236372 | 1^st^ > 2^nd^ |
| FRC1 north - FRC1 south | 0.084006 | -0.30858 | 0.449011 | na |
| FRC1 north - Wax south | -0.92327 | -1.31585 | -0.55187 | 1^st^ < 2^nd^ |
| Wax north - Control south | -1.28448 | -1.67924 | -0.92462 | 1^st^ < 2^nd^ |
| Wax north - FRC2 south | 1.94211 | 1.546801 | 2.322118 | 1^st^ > 2^nd^ |
| Wax north - FRC1 south | 1.147715 | 0.772227 | 1.552353 | 1^st^ > 2^nd^ |
| Wax north - Wax south | 0.144615 | -0.24718 | 0.513839 | na |
| Control south - FRC2 south | 3.223637 | 2.915866 | 3.555587 | 1^st^ > 2^nd^ |
| Control south - FRC1 south | 2.442069 | 2.149176 | 2.723721 | 1^st^ > 2^nd^ |
| Control south - Wax south | 1.428296 | 1.142517 | 1.713669 | 1^st^ > 2^nd^ |
| FRC2 south - FRC1 south | -0.78119 | -1.10239 | -0.50657 | 1^st^ < 2^nd^ |
| FRC2 south - Wax south | -1.80044 | -2.08308 | -1.47334 | 1^st^ < 2^nd^ |
| FRC1 south - Wax south | -1.01586 | -1.2686 | -0.72316 | 1^st^ < 2^nd^ |

**Table S2.10** Mean values of fouling categories for the core of the devices across core treatments, sites and experimental timepoints.

|  |  | Control | | | | | Wax | | | | | FRC1 | | | | | FRC2 | | | | |
| --- | --- | --- | --- | --- | --- | --- | --- | --- | --- | --- | --- | --- | --- | --- | --- | --- | --- | --- | --- | --- | --- |
|  |  | t=0 | t=8 | t=20 | t=30 | t=46 | t=0 | t=8 | t=20 | t=30 | t=46 | t=0 | t=8 | t=20 | t=30 | t=46 | t=0 | t=8 | t=20 | t=30 | t=46 |
| North | clear | 100.0 | 8.3 | 1.9 | 0.6 | 0.7 | 100.0 | 28.3 | 20.4 | 2.8 | 2.6 | 100.0 | 42.2 | 24.7 | 18.2 | 18.5 | 100.0 | 42.3 | 34.7 | 16.1 | 15.8 |
|  | coral | 0.0 | 0.0 | 6.5 | 13.4 | 13.4 | 0.0 | 0.0 | 2.8 | 8.2 | 8.7 | 0.0 | 0.1 | 4.5 | 6.1 | 4.8 | 0.0 | 0.0 | 2.7 | 11.1 | 11.3 |
|  | fouling_all | 0.0 | 87.6 | 85.8 | 69.4 | 69.4 | 0.0 | 66.8 | 72.1 | 69.2 | 68.0 | 0.0 | 52.0 | 62.2 | 58.3 | 59.2 | 0.0 | 51.9 | 50.7 | 56.2 | 56.1 |
|  | other | 0.0 | 4.1 | 5.7 | 16.7 | 16.6 | 0.0 | 4.9 | 4.7 | 19.8 | 20.6 | 0.0 | 5.8 | 8.6 | 17.4 | 17.4 | 0.0 | 5.8 | 11.9 | 16.5 | 16.9 |
| Central | clear | 100.0 | 2.8 | 4.0 | 2.4 | 1.0 | 100.0 | 26.0 | 10.7 | 10.4 | 9.7 | 100.0 | 44.1 | 29.7 | 17.6 | 15.3 | 100.0 | 41.3 | 37.0 | 30.8 | 30.8 |
|  | coral | 0.0 | 0.0 | 1.2 | 7.9 | 10.4 | 0.0 | 0.0 | 1.3 | 3.7 | 6.2 | 0.0 | 0.0 | 2.4 | 7.6 | 9.1 | 0.0 | 0.0 | 1.7 | 6.0 | 7.5 |
|  | fouling_all | 0.0 | 93.1 | 87.0 | 72.7 | 74.1 | 0.0 | 72.0 | 77.3 | 65.1 | 67.0 | 0.0 | 48.9 | 56.5 | 55.9 | 59.0 | 0.0 | 51.0 | 51.4 | 48.1 | 52.2 |
|  | other | 0.0 | 4.2 | 7.8 | 17.0 | 14.4 | 0.0 | 2.0 | 10.7 | 20.8 | 17.1 | 0.0 | 7.0 | 11.4 | 19.0 | 16.6 | 0.0 | 7.7 | 10.0 | 15.1 | 9.4 |
| South | clear | 100.0 | 10.7 | 4.1 | 2.8 | 2.8 | 100.0 | 39.7 | 18.8 | 7.5 | 7.5 | 100.0 | 45.4 | 35.1 | 23.9 | 23.9 | 100.0 | 48.8 | 42.5 | 33.0 | 32.8 |
|  | coral | 0.0 | 0.0 | 3.9 | 10.8 | 10.8 | 0.0 | 0.0 | 3.2 | 6.5 | 6.5 | 0.0 | 0.0 | 2.2 | 5.8 | 5.8 | 0.0 | 0.0 | 2.7 | 8.6 | 8.4 |
|  | fouling_all | 0.0 | 88.3 | 88.8 | 79.4 | 79.3 | 0.0 | 58.0 | 72.4 | 76.0 | 76.0 | 0.0 | 51.7 | 59.8 | 60.2 | 60.2 | 0.0 | 49.9 | 52.3 | 51.3 | 51.3 |
|  | other | 0.0 | 1.0 | 3.1 | 7.0 | 7.0 | 0.0 | 2.2 | 5.6 | 10.0 | 10.0 | 0.0 | 2.8 | 2.9 | 10.1 | 10.1 | 0.0 | 1.2 | 2.6 | 7.1 | 7.4 |


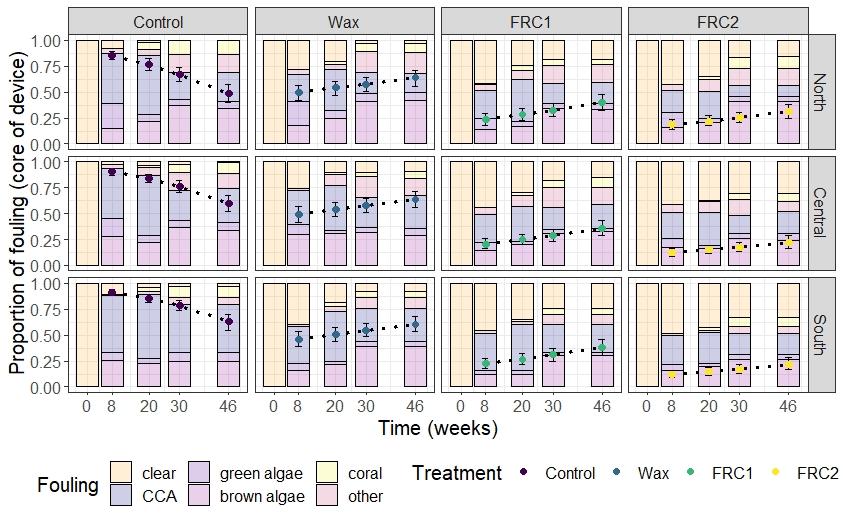


**Figure S2.1** Fitted model predictions for fouling of device (core only) deployed at three different locations at Davies Reef. Colours represent the four different treatments as well fouling categories. Bar plots on the background represent the total proportion of individual fouling categories within each treatment, timepoint and site.

**
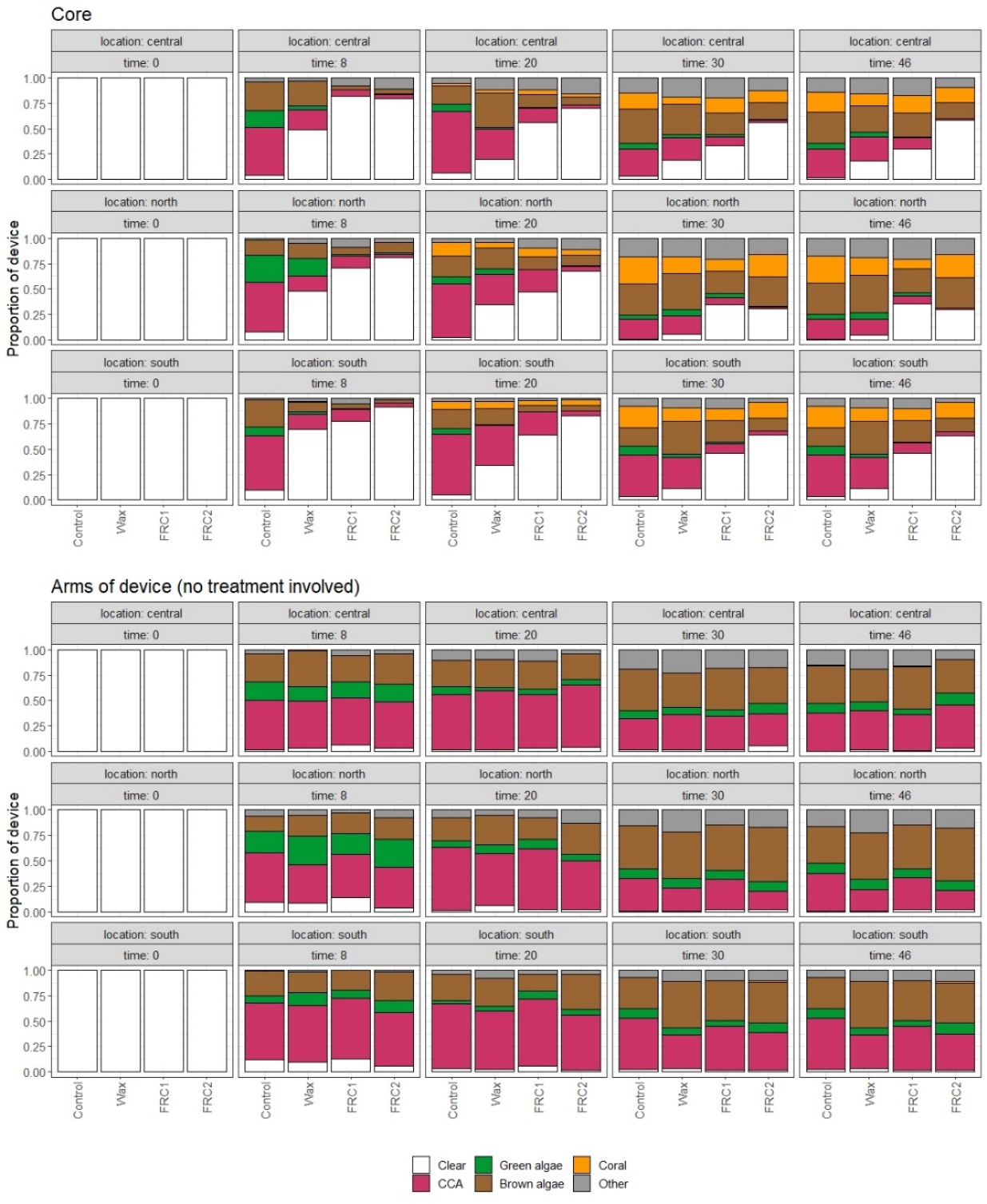
**

**Figure S2.2** Proportion of the area of device (core or arms) colonised by the different fouling categories, including additional categories (clean, coral and other) relative to treatment, sites and timepoints.

**
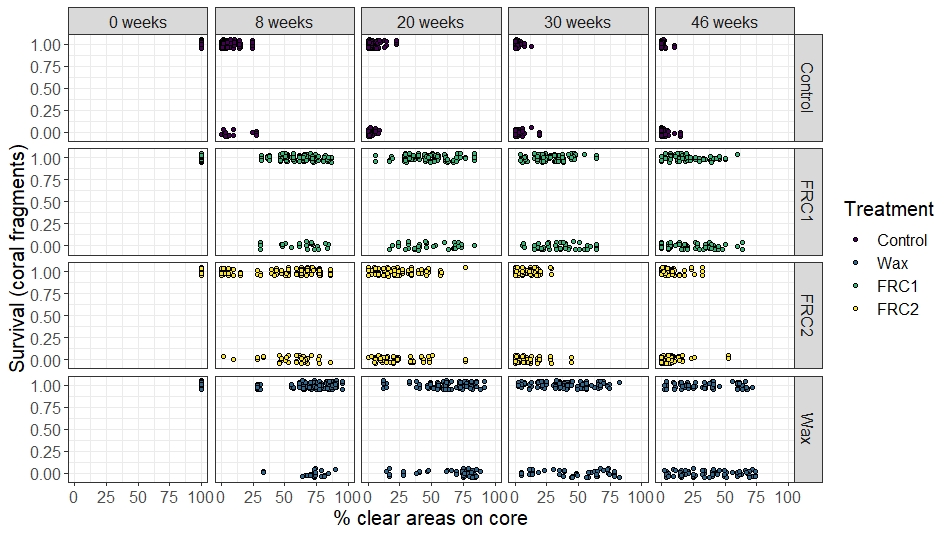
**

**Figure S2.3** Survival proportion of coral microfragments in relation to the proportion of clear areas (non-fouled) of the core of the device relative to treatment and timepoints.

**Table S2.11** Pairwise comparison of survival estimates (mod#7) according to treatment and location. Light grey rows showing the 1^st^ group having higher survival than the 2^nd^, dark grey showing 1^st^ group having lower survival than the 2^nd^, and “na” showing no difference between groups.

| Contrast (1^st^ vs 2^nd^) | estimate | lower.HPD | upper.HPD | difference |
| --- | --- | --- | --- | --- |
| Control south - FRC2 south | -0.63582 | -1.727 | 0.504173 | na |
| Control south - FRC1 south | 0.939303 | -0.20895 | 1.991964 | na |
| Control south - Wax south | 1.402601 | 0.300736 | 2.50763 | 1^st^ > 2^nd^ |
| Control south - Control central | -0.44035 | -2.13342 | 1.000158 | na |
| Control south - FRC2 central | 0.777495 | -0.81799 | 2.199338 | na |
| Control south - FRC1 central | -0.21948 | -1.80049 | 1.313925 | na |
| Control south - Wax central | 1.367711 | -0.05812 | 2.974665 | na |
| Control south - Control north | -1.20242 | -2.84134 | 0.412022 | na |
| Control south - FRC2 north | -0.52047 | -2.1035 | 0.982835 | na |
| Control south - FRC1 north | -0.63282 | -2.23029 | 0.957771 | na |
| Control south - Wax north | 0.005285 | -1.58533 | 1.484757 | na |
| FRC2 south - FRC1 south | 1.567516 | 0.511943 | 2.673475 | 1^st^ > 2^nd^ |
| FRC2 south - Wax south | 2.040201 | 0.93964 | 3.177642 | 1^st^ > 2^nd^ |
| FRC2 south - Control central | 0.160116 | -1.31844 | 1.80954 | na |
| FRC2 south - FRC2 central | 1.421002 | 0.039781 | 3.047444 | 1^st^ > 2^nd^ |
| FRC2 south - FRC1 central | 0.396428 | -1.12802 | 1.938062 | na |
| FRC2 south - Wax central | 2.003622 | 0.500228 | 3.536795 | 1^st^ > 2^nd^ |
| FRC2 south - Control north | -0.58946 | -2.16691 | 1.06583 | na |
| FRC2 south - FRC2 north | 0.091906 | -1.37903 | 1.746908 | na |
| FRC2 south - FRC1 north | 0.014912 | -1.68714 | 1.460518 | na |
| FRC2 south - Wax north | 0.639182 | -1.01483 | 2.209531 | na |
| FRC1 south - Wax south | 0.465509 | -0.61105 | 1.49229 | na |
| FRC1 south - Control central | -1.39353 | -2.87179 | 0.145313 | na |
| FRC1 south - FRC2 central | -0.14634 | -1.51761 | 1.444244 | na |
| FRC1 south - FRC1 central | -1.15104 | -2.66266 | 0.321699 | na |
| FRC1 south - Wax central | 0.425845 | -1.01955 | 1.816404 | na |
| FRC1 south - Control north | -2.15452 | -3.66419 | -0.52468 | 1^st^ < 2^nd^ |
| FRC1 south - FRC2 north | -1.46682 | -2.9761 | 0.049394 | na |
| FRC1 south - FRC1 north | -1.56851 | -3.03368 | -0.01267 | na |
| FRC1 south - Wax north | -0.92775 | -2.3996 | 0.612841 | na |
| Wax south - Control central | -1.87178 | -3.43704 | -0.38068 | 1^st^ < 2^nd^ |
| Wax south - FRC2 central | -0.60976 | -2.18419 | 0.8294 | na |
| Wax south - FRC1 central | -1.62712 | -3.22802 | -0.10611 | 1^st^ < 2^nd^ |
| Wax south - Wax central | -0.04563 | -1.50398 | 1.445155 | na |
| Wax south - Control north | -2.61236 | -4.2019 | -1.03089 | 1^st^ < 2^nd^ |
| Wax south - FRC2 north | -1.92478 | -3.36893 | -0.31028 | 1^st^ < 2^nd^ |
| Wax south - FRC1 north | -2.01684 | -3.61745 | -0.543 | 1^st^ < 2^nd^ |
| Wax south - Wax north | -1.40466 | -2.92625 | 0.161514 | na |
| Control central - FRC2 central | 1.264997 | 0.22992 | 2.354189 | 1^st^ > 2^nd^ |
| Control central - FRC1 central | 0.230793 | -0.9166 | 1.285824 | na |
| Control central - Wax central | 1.82957 | 0.816176 | 2.974905 | 1^st^ > 2^nd^ |
| Control central - Control north | -0.72936 | -2.31058 | 0.748886 | na |
| Control central - FRC2 north | -0.07144 | -1.61745 | 1.444898 | na |
| Control central - FRC1 north | -0.15279 | -1.70726 | 1.336232 | na |
| Control central - Wax north | 0.470804 | -1.01689 | 2.082674 | na |
| FRC2 central - FRC1 central | -1.02085 | -2.22217 | -0.07518 | 1^st^ < 2^nd^ |
| FRC2 central - Wax central | 0.581181 | -0.38411 | 1.6036 | na |
| FRC2 central - Control north | -2.00296 | -3.54183 | -0.38869 | 1^st^ < 2^nd^ |
| FRC2 central - FRC2 north | -1.31164 | -2.85793 | 0.17015 | na |
| FRC2 central - FRC1 north | -1.40731 | -2.90836 | 0.061755 | na |
| FRC2 central - Wax north | -0.80172 | -2.3422 | 0.71659 | na |
| FRC1 central - Wax central | 1.591085 | 0.583449 | 2.671116 | 1^st^ > 2^nd^ |
| FRC1 central - Control north | -0.97396 | -2.52606 | 0.558292 | na |
| FRC1 central - FRC2 north | -0.29929 | -1.82182 | 1.255488 | na |
| FRC1 central - FRC1 north | -0.40729 | -1.94943 | 1.12713 | na |
| FRC1 central - Wax north | 0.243566 | -1.35144 | 1.775134 | na |
| Wax central - Control north | -2.56696 | -4.1438 | -1.04379 | 1^st^ < 2^nd^ |
| Wax central - FRC2 north | -1.87634 | -3.4872 | -0.47553 | 1^st^ < 2^nd^ |
| Wax central - FRC1 north | -1.97794 | -3.53654 | -0.55812 | 1^st^ < 2^nd^ |
| Wax central - Wax north | -1.36944 | -2.93049 | 0.127265 | na |
| Control north - FRC2 north | 0.692542 | -0.50293 | 1.991477 | na |
| Control north - FRC1 north | 0.591165 | -0.62035 | 1.853131 | na |
| Control north - Wax north | 1.212197 | 0.009648 | 2.440148 | 1^st^ > 2^nd^ |
| FRC2 north - FRC1 north | -0.09139 | -1.18044 | 1.071019 | na |
| FRC2 north - Wax north | 0.509995 | -0.59641 | 1.630295 | na |
| FRC1 north - Wax north | 0.617235 | -0.49301 | 1.733073 | na |

**Table S2.12** Permanova for the comparison of benthic community data across sites and transects. Matrix of PCOA base on Bray distances.

|  | DF | SumsOfSqs | MeansSqs | F.Model | R2 | Pr(>F) | Sig |
| --- | --- | --- | --- | --- | --- | --- | --- |
| Site | 2 | 0.507 | 0.25335 | 3.6204 | 0.00708 | 0.003 | ** |
| Transect | 33 | 15.394 | 0.46649 | 6.6663 | 0.21499 | 0.001 | *** |
| Residuals | 796 | 55.703 | 0.06998 |  | 0.77793 |  |  |
| Total | 831 | 71.603 |  |  | 1.00000 |  |  |

*Signif. codes: 0 ‘***’ 0.001 ‘**’ 0.01 ‘*’ 0.05 ‘.’ 0.1 ‘ ’ 1*

**Table S2.13** Pairwise comparisons between pairs of deployment sites.

| pairs | DF | SumsOfSqs | F.Model | R2 | p.value | p.adjusted sig |
| --- | --- | --- | --- | --- | --- | --- |
| Davies North vs Davies Central | 1 | 0.2831148 | 3.418537 | 0.006121819 | 0.019 | 0.057 |
| Davies North vs Davies South | 1 | 0.1959441 | 2.185017 | 0.003971423 | 0.120 | 0.360 |
| Davies Central vs Davies South | 1 | 0.2802595 | 3.303318 | 0.005916709 | 0.017 | 0.051 |

**Table S2.14** Proportion of benthic categories observed on each location across experimental timepoints.

**Table S2.15** Proportion of benthic categories observed on each location across experimental timepoints.

|  |  | Time | | | |
| --- | --- | --- | --- | --- | --- |
|  |  | **t=8** | **t=20** | **t=30** | **t=46** |
| North | coral | 6.99 | 8.81 | 8.09 | 8.53 |
|  | macroalgae | 14.32 | 11.41 | 13.05 | 14.90 |
|  | recently killed coral | 0.00 | 0.00 | 0.00 | 0.00 |
|  | rock with CCA | 18.24 | 37.68 | 22.92 | 20.45 |
|  | rock with turf algae | 57.28 | 36.30 | 51.72 | 52.12 |
|  | rubble with CCA | 0.24 | 0.03 | 0.21 | 0.47 |
|  | rubble with turf algae | 0.42 | 0.00 | 0.00 | 0.09 |
|  | sand | 0.07 | 0.06 | 0.06 | 0.00 |
|  | silt | 2.01 | 5.18 | 3.49 | 3.22 |
|  | soft coral | 0.25 | 0.23 | 0.20 | 0.11 |
|  | sponge | 0.19 | 0.29 | 0.26 | 0.06 |
| Central | coral | 12.9973 | 15.625 | 15.9538 | 16.7977 |
|  | macroalgae | 5.64223 | 4.93461 | 7.06375 | 4.93994 |
|  | recently killed coral | 0 | 0 | 0 | 0 |
|  | rock with cca | 28.6555 | 27.7819 | 22.8155 | 27.4159 |
|  | rock with turf algae | 32.0293 | 34.8668 | 47.6309 | 40.9391 |
|  | rubble with cca | 0.59492 | 0.36425 | 0.08992 | 0.52837 |
|  | rubble with turf algae | 0.03157 | 0.05809 | 0 | 0.09066 |
|  | sand | 0.2027 | 0.12552 | 0.03322 | 0.0961 |
|  | silt | 17.7856 | 14.6113 | 5.34778 | 8.51956 |
|  | soft coral | 0.8639 | 0.62919 | 0.28093 | 0.29324 |
|  | sponge | 0.67581 | 0.97402 | 0.75165 | 0.37942 |
| South | coral | 16.1768 | 14.9654 | 14.1704 | 13.5611 |
|  | macroalgae | 8.33916 | 6.1134 | 7.12683 | 5.83528 |
|  | recently killed coral | 0 | 0 | 0 | 0 |
|  | rock with cca | 28.8098 | 36.1275 | 23.7394 | 26.8288 |
|  | rock with turf algae | 38.6247 | 32.1741 | 49.3051 | 48.6356 |
|  | rubble with cca | 0.47134 | 1.44984 | 0.8061 | 0.74385 |
|  | rubble with turf algae | 0.0323 | 0.02857 | 0.03377 | 0.08992 |
|  | sand | 0.23452 | 0.03106 | 0 | 0.03484 |
|  | silt | 5.84765 | 8.06705 | 3.89919 | 3.25329 |
|  | soft coral | 1.17293 | 0.53536 | 0.60136 | 0.61522 |
|  | sponge | 0.2592 | 0.4729 | 0.28413 | 0.37289 |

**Table S2.16** Pairwise comparison of survival estimates in correlation to invertebrate as benthic category (mod#4b) according to treatment and location. Light grey rows showing the 1^st^ group having higher survival than the 2^nd^, dark grey showing 1^st^ group having lower survival than the 2^nd^, and “na” showing no difference between groups.

| Contrast (1^st^ vs 2^nd^) | estimate | lower.HPD | upper.HPD | difference |
| --- | --- | --- | --- | --- |
| Control central - FRC2 central | 1.155002 | -0.07883 | 2.362114 | na |
| Control central - FRC1 central | 0.210583 | -1.06493 | 1.405543 | na |
| Control central - Wax central | 1.708998 | 0.627958 | 3.046755 | 1^st^ > 2^nd^ |
| Control central - Control north | -0.31266 | -2.02226 | 1.316002 | na |
| Control central - FRC2 north | 0.41232 | -1.09188 | 1.97912 | na |
| Control central - FRC1 north | 0.079295 | -1.47466 | 1.715953 | na |
| Control central - Wax north | 0.859998 | -0.70925 | 2.442023 | na |
| Control central - Control south | 0.515952 | -0.99185 | 2.108622 | na |
| Control central - FRC2 south | 0.051496 | -1.54262 | 1.587802 | na |
| Control central - FRC1 south | 1.429556 | -0.17449 | 2.993002 | na |
| Control central - Wax south | 2.188097 | 0.667539 | 3.803096 | 1^st^ > 2^nd^ |
| FRC2 central - FRC1 central | -0.94365 | -2.15453 | 0.252764 | na |
| FRC2 central - Wax central | 0.550822 | -0.71654 | 1.665209 | na |
| FRC2 central - Control north | -1.44767 | -3.00818 | 0.21421 | na |
| FRC2 central - FRC2 north | -0.71829 | -2.27021 | 0.801884 | na |
| FRC2 central - FRC1 north | -1.07749 | -2.54052 | 0.510629 | na |
| FRC2 central - Wax north | -0.29309 | -1.88171 | 1.240917 | na |
| FRC2 central - Control south | -0.62151 | -2.23838 | 0.846857 | na |
| FRC2 central - FRC2 south | -1.11983 | -2.69567 | 0.469717 | na |
| FRC2 central - FRC1 south | 0.2748 | -1.33598 | 1.791274 | na |
| FRC2 central - Wax south | 1.041077 | -0.4853 | 2.58777 | na |
| FRC1 central - Wax central | 1.487607 | 0.298863 | 2.721988 | 1^st^ > 2^nd^ |
| FRC1 central - Control north | -0.52045 | -2.09316 | 1.100909 | na |
| FRC1 central - FRC2 north | 0.214151 | -1.29957 | 1.701509 | na |
| FRC1 central - FRC1 north | -0.14084 | -1.7961 | 1.312556 | na |
| FRC1 central - Wax north | 0.645981 | -0.92126 | 2.164693 | na |
| FRC1 central - Control south | 0.303002 | -1.11013 | 1.914666 | na |
| FRC1 central - FRC2 south | -0.16634 | -1.67188 | 1.394608 | na |
| FRC1 central - FRC1 south | 1.213024 | -0.27991 | 2.792343 | na |
| FRC1 central - Wax south | 1.952274 | 0.51587 | 3.578943 | 1^st^ > 2^nd^ |
| Wax central - Control north | -2.00214 | -3.70726 | -0.39615 | 1^st^ < 2^nd^ |
| Wax central - FRC2 north | -1.28631 | -2.93707 | 0.091289 | na |
| Wax central - FRC1 north | -1.61897 | -3.21351 | -0.22251 | 1^st^ < 2^nd^ |
| Wax central - Wax north | -0.85995 | -2.35227 | 0.796121 | na |
| Wax central - Control south | -1.19293 | -2.87335 | 0.251124 | na |
| Wax central - FRC2 south | -1.67497 | -3.19805 | -0.17085 | 1^st^ < 2^nd^ |
| Wax central - FRC1 south | -0.2867 | -1.83193 | 1.263185 | na |
| Wax central - Wax south | 0.47319 | -1.11887 | 1.976917 | na |
| Control north - FRC2 north | 0.728767 | -0.60326 | 1.978865 | na |
| Control north - FRC1 north | 0.393486 | -0.93708 | 1.696113 | na |
| Control north - Wax north | 1.159272 | -0.07976 | 2.592364 | na |
| Control north - Control south | 0.814154 | -0.80442 | 2.464268 | na |
| Control north - FRC2 south | 0.34269 | -1.24115 | 1.956292 | na |
| Control north - FRC1 south | 1.728039 | 0.16553 | 3.419384 | 1^st^ > 2^nd^ |
| Control north - Wax south | 2.498371 | 0.997654 | 4.201161 | 1^st^ > 2^nd^ |
| FRC2 north - FRC1 north | -0.33471 | -1.64716 | 0.861132 | na |
| FRC2 north - Wax north | 0.444371 | -0.82146 | 1.640063 | na |
| FRC2 north - Control south | 0.093512 | -1.45424 | 1.578206 | na |
| FRC2 north - FRC2 south | -0.38669 | -1.87125 | 1.089787 | na |
| FRC2 north - FRC1 south | 0.997571 | -0.53662 | 2.542787 | na |
| FRC2 north - Wax south | 1.788806 | 0.340276 | 3.345966 | 1^st^ > 2^nd^ |
| FRC1 north - Wax north | 0.790402 | -0.44394 | 2.129252 | na |
| FRC1 north - Control south | 0.443019 | -1.18235 | 2.015554 | na |
| FRC1 north - FRC2 south | -0.0353 | -1.48089 | 1.590307 | na |
| FRC1 north - FRC1 south | 1.352493 | -0.23935 | 2.90066 | na |
| FRC1 north - Wax south | 2.121844 | 0.614882 | 3.756299 | 1^st^ > 2^nd^ |
| Wax north - Control south | -0.35283 | -1.96389 | 1.205212 | na |
| Wax north - FRC2 south | -0.81493 | -2.36747 | 0.754962 | na |
| Wax north - FRC1 south | 0.548671 | -1.08654 | 2.092105 | na |
| Wax north - Wax south | 1.342001 | -0.28421 | 2.918863 | na |
| Control south - FRC2 south | -0.48424 | -1.70046 | 0.804075 | na |
| Control south - FRC1 south | 0.904977 | -0.35363 | 2.137713 | na |
| Control south - Wax south | 1.693678 | 0.465398 | 3.015911 | 1^st^ > 2^nd^ |
| FRC2 south - FRC1 south | 1.373411 | 0.076469 | 2.616685 | 1^st^ > 2^nd^ |
| FRC2 south - Wax south | 2.156032 | 0.892969 | 3.411254 | 1^st^ > 2^nd^ |
| FRC1 south - Wax south | 0.775089 | -0.4329 | 2.010635 | na |

**Table S2.17** Pairwise comparison of survival estimates in correlation to turf-macroalgae as benthic category (mod#1b) according to treatment and location. Light grey rows showing the 1^st^ group having higher survival than the 2^nd^, dark grey showing 1^st^ group having lower survival than the 2^nd^, and “na” showing no difference between groups.

| Contrast (1^st^ vs 2^nd^) | estimate | lower.HPD | upper.HPD | difference |
| --- | --- | --- | --- | --- |
| Control central - FRC2 central | 1.191276 | -0.06138 | 2.455154 | na |
| Control central - FRC1 central | 0.15916 | -1.05965 | 1.382106 | na |
| Control central - Wax central | 1.699866 | 0.487033 | 2.933351 | 1^st^ > 2^nd^ |
| Control central - Control north | -0.39091 | -2.06888 | 1.333728 | na |
| Control central - FRC2 north | 0.392449 | -1.19227 | 1.949327 | na |
| Control central - FRC1 north | 0.102769 | -1.53124 | 1.731013 | na |
| Control central - Wax north | 0.78295 | -0.77594 | 2.458622 | na |
| Control central - Control south | 0.518461 | -1.08233 | 2.105016 | na |
| Control central - FRC2 south | -0.00743 | -1.58233 | 1.589547 | na |
| Control central - FRC1 south | 1.491472 | -0.10732 | 3.057309 | na |
| Control central - Wax south | 2.133672 | 0.581136 | 3.806043 | 1^st^ > 2^nd^ |
| FRC2 central - FRC1 central | -1.02666 | -2.29801 | 0.180187 | na |
| FRC2 central - Wax central | 0.534102 | -0.7545 | 1.66225 | na |
| FRC2 central - Control north | -1.55563 | -3.20579 | 0.123022 | na |
| FRC2 central - FRC2 north | -0.79267 | -2.34438 | 0.7851 | na |
| FRC2 central - FRC1 north | -1.07862 | -2.60584 | 0.5847 | na |
| FRC2 central - Wax north | -0.39828 | -1.94128 | 1.154699 | na |
| FRC2 central - Control south | -0.65974 | -2.23787 | 0.969268 | na |
| FRC2 central - FRC2 south | -1.18399 | -2.70521 | 0.391636 | na |
| FRC2 central - FRC1 south | 0.318198 | -1.3085 | 1.9613 | na |
| FRC2 central - Wax south | 0.950887 | -0.60967 | 2.539549 | na |
| FRC1 central - Wax central | 1.550972 | 0.340008 | 2.772491 | 1^st^ > 2^nd^ |
| FRC1 central - Control north | -0.5491 | -2.20498 | 1.062469 | na |
| FRC1 central - FRC2 north | 0.233703 | -1.36751 | 1.754971 | na |
| FRC1 central - FRC1 north | -0.07002 | -1.69607 | 1.518907 | na |
| FRC1 central - Wax north | 0.646859 | -0.8517 | 2.280656 | na |
| FRC1 central - Control south | 0.373921 | -1.22982 | 1.92668 | na |
| FRC1 central - FRC2 south | -0.15026 | -1.88203 | 1.321571 | na |
| FRC1 central - FRC1 south | 1.323583 | -0.22925 | 2.916669 | na |
| FRC1 central - Wax south | 1.967797 | 0.460628 | 3.505632 | 1^st^ > 2^nd^ |
| Wax central - Control north | -2.08591 | -3.67842 | -0.42632 | 1^st^ < 2^nd^ |
| Wax central - FRC2 north | -1.3126 | -2.92694 | 0.151664 | na |
| Wax central - FRC1 north | -1.61475 | -3.20962 | -0.00872 | 1^st^ < 2^nd^ |
| Wax central - Wax north | -0.92516 | -2.45052 | 0.622028 | na |
| Wax central - Control south | -1.17697 | -2.69687 | 0.432429 | na |
| Wax central - FRC2 south | -1.70998 | -3.25546 | -0.16417 | 1^st^ < 2^nd^ |
| Wax central - FRC1 south | -0.19855 | -1.69109 | 1.418477 | na |
| Wax central - Wax south | 0.424697 | -1.04735 | 2.031383 | na |
| Control north - FRC2 north | 0.770576 | -0.47575 | 2.057572 | na |
| Control north - FRC1 north | 0.486675 | -0.76255 | 1.845787 | na |
| Control north - Wax north | 1.172142 | -0.15282 | 2.490797 | na |
| Control north - Control south | 0.913611 | -0.67662 | 2.617718 | na |
| Control north - FRC2 south | 0.389636 | -1.22164 | 2.042758 | na |
| Control north - FRC1 south | 1.881059 | 0.207052 | 3.586336 | 1^st^ > 2^nd^ |
| Control north - Wax south | 2.523542 | 0.903655 | 4.195163 | 1^st^ > 2^nd^ |
| FRC2 north - FRC1 north | -0.28067 | -1.51296 | 0.923753 | na |
| FRC2 north - Wax north | 0.414137 | -0.852 | 1.634876 | na |
| FRC2 north - Control south | 0.142021 | -1.46324 | 1.733434 | na |
| FRC2 north - FRC2 south | -0.39469 | -1.95043 | 1.25135 | na |
| FRC2 north - FRC1 south | 1.095365 | -0.51634 | 2.605774 | na |
| FRC2 north - Wax south | 1.725467 | 0.16149 | 3.322113 | 1^st^ > 2^nd^ |
| FRC1 north - Wax north | 0.701063 | -0.50637 | 1.995155 | na |
| FRC1 north - Control south | 0.428615 | -1.14824 | 2.001518 | na |
| FRC1 north - FRC2 south | -0.10044 | -1.59583 | 1.534369 | na |
| FRC1 north - FRC1 south | 1.376412 | -0.24013 | 2.942299 | na |
| FRC1 north - Wax south | 2.018211 | 0.486916 | 3.577421 | 1^st^ > 2^nd^ |
| Wax north - Control south | -0.2588 | -1.8815 | 1.353669 | na |
| Wax north - FRC2 south | -0.79669 | -2.40463 | 0.740241 | na |
| Wax north - FRC1 south | 0.693273 | -0.92863 | 2.208881 | na |
| Wax north - Wax south | 1.343257 | -0.34422 | 2.868537 | na |
| Control south - FRC2 south | -0.5131 | -1.72211 | 0.81197 | na |
| Control south - FRC1 south | 0.979118 | -0.26491 | 2.218572 | na |
| Control south - Wax south | 1.607756 | 0.349444 | 2.867405 | 1^st^ > 2^nd^ |
| FRC2 south - FRC1 south | 1.488819 | 0.302846 | 2.756525 | 1^st^ > 2^nd^ |
| FRC2 south - Wax south | 2.145071 | 0.916131 | 3.357392 | 1^st^ > 2^nd^ |
| FRC1 south - Wax south | 0.653832 | -0.51409 | 1.941152 | na |
